# Evaluation of intracavitary administration of curcumin for the treatment of sarcomatoid mesothelioma

**DOI:** 10.18632/oncotarget.15744

**Published:** 2017-02-25

**Authors:** Daniel L. Pouliquen, Béatrice Nawrocki-Raby, Joëlle Nader, Stéphanie Blandin, Myriam Robard, Philippe Birembaut, Marc Grégoire

**Affiliations:** ^1^ INSERM, UMR 1232, Nantes, France; ^2^ Université de Nantes, Nantes, France; ^3^ CNRS ERL, Nantes, France; ^4^ INSERM, UMR-S 903, Reims, France; ^5^ Université de Reims Champagne-Ardenne, Reims, France; ^6^ SFR CAP-Santé, Reims, France; ^7^ Plate-forme MicroPICell, SFR François Bonamy, Nantes, France; ^8^ Laboratory of Biopathology, CHU Reims, Reims, France

**Keywords:** curcumin, sarcomatoid mesothelioma, rat, orthotopic tumor model

## Abstract

A rat model of sarcomatoid mesothelioma, mimicking some of the worst clinical conditions encountered, was established to evaluate the therapeutic potential of intracavitary curcumin administration.

The M5-T1 cell line, selected from a collection established from F344 rats induced with asbestos, produces tumors within three weeks, with extended metastasis in normal tissues, after intraperitoneal inoculation in syngeneic rats. The optimal concentration/time conditions for killing M5-T1 cells with curcumin were first determined *in vitro*. Secondly, the potential of intraperitoneal curcumin administration to kill tumor cells *in vivo* was evaluated in tumor-bearing rats, in comparison with a reference epigenetic drug, SAHA.

Both agents administered at days 21 and 26 after tumor challenge produced necrosis within the solid tumors at day 28. However, tumor tissue necrosis induced with curcumin was much more extensive than with SAHA, and was characterized by infiltration with mononuclear phagocytic cells. In contrast, tumor tissue treated with SAHA contained foci of resistant cells and was infiltrated by many isolated CD8+ cells. The treatment of tumor-bearing rats with 1.5 mg/kg curcumin on days 7, 9, 11 and 14 after tumor challenge dramatically reduced the mean total tumor mass at day 16*.* Clusters of CD8+ T lymphocytes were observed at the periphery of small residual tumor masses in the peritoneal cavity, which presented a significant reduction in mitotic index, IL6 and vimentin expression compared with tumors in untreated rats.

These data open up interesting new prospects for the therapy of sarcomatoid mesothelioma with curcumin and its derivatives.

## INTRODUCTION

Malignant mesothelioma (MM) is one of the worst cancers in terms of clinical outcome. The sarcomatoid type is the most aggressive of the three major histological subtypes, with a median survival of 3.5 months [1]. Despite some advances in chemotherapy with the association of cisplatin and pemetrexed [2], in over a decade there has been no significant progress in systemic treatment for MM [3], and this depressing situation is mainly due to the to a lack of understanding of the complex biology of this cancer [4]. The situation is even more complicated by the fact that MM is often detected late in the development of the disease, with patients presenting with advanced cancers associated with breathlessness and pain [5]. Patients with sarcomatoid MM or those who are too ill to tolerate treatment are frequently excluded from therapeutic approaches, while medical complications are encountered in patients with peritoneal MM [6] or when the diaphragm is affected [7]. Thus, nearly two-thirds of patients fail to show a response to current procedures. To improve outcomes for patients with such aggressive cancers, clinicians are faced with two challenges. The first is to reduce tumor development and its wasting consequences with a procedure that does not develop resistance to treatment, and with limited toxicity. The second is to stimulate recognition of tumor cells by induction of a specific immune response directed towards eradicating residual tumor cells and avoiding early relapses.

To improve tumor cell recognition by immune cells, the last decade has confirmed the potential of epigenetic drugs in reactivating the expression of silenced genes [8]. We have previously demonstrated that, in addition to cell cytoxicity, treatment with SAHA (Vorinostat^®^) contributes to an increase in the immunogenicity of tumor cells *in vivo* [9]. In contrast, the first goal stated above appears to be much more difficult to reach. Nevertheless, both the literature and clinical trials have confirmed the potential of a natural compound, curcumin, against various types of cancers [10, 11]. Six years ago, the epigenetic modulation of target genes by this molecule was also highlighted [12], emphasizing the interest of drugs relevant to polypharmacology [13], as opposed to targeted therapies. Subsequently, the modulation of DNA methylation, histone modification and microRNAs by curcumin have been reviewed [14]. To date, besides its evaluation as a histone deacetylase inhibitor (HDACi) in clinical trials for lymphoma therapy [15], curcumin, alone [16] or in combination with other natural compounds [17], has been used mainly for chemoprevention as an epigenetic diet. However, this agent can also be selected for therapy, in particular using other routes of administration than the diet.

To address the poor bioavailability of curcumin, a first strategy has been based on the design of various curcumin derivatives, analogs and prodrugs that exhibit enhanced water solubility and biological activities [18, 19]. A second strategy has consisted of injection of curcumin-loaded nanocarriers to increase therapeutic curcumin concentration at the target site and to avoid extensive metabolism by the liver. For this purpose, polymeric and albumin nanoparticles have been tested as carriers in normal mice and rats, demonstrating that encapsulation of curcumin improves pharmacokinetic parameters. Curcumin-loaded liposomes, albumin or polymeric nanoparticles have also been evaluated on different types of xenograft tumor models in athymic mice [19]. However, the pertinence of xenografts grown in immunodeficient strains of mice for predicting therapeutic efficacy in patients raises a number of questions [20] and consequently orthotopic tumor models are now preferred [21]. Finally, a third strategy has consisted of intraperitoneal (i.p.) administration of curcumin or curcumin-loaded nanocarriers. The benefit provided by the use of this route of administration, compared with systemic chemotherapy, has already been documented for the treatment of cancers with peritoneal dissemination [22]. Since a pioneering study on a rat histiocytic tumor transplanted i.p. in an outbred rat strain was performed [23], this strategy has been successfully used for the treatment of C6 rat glioblastomas implanted in Wistar rats, first using free curcumin and then after the administration of curcumin incorporated into lipid-core nanocapsules [24].

Applied to the treatment of mesothelioma, we first demonstrated that curcumin efficiently kills murine MM cells *in vitro* [9]. Subsequently, this observation was confirmed by several independent studies on different murine and human MM cell lines [25–28]. In the present study, we have evaluated the therapeutic potential of this molecule *in vivo* in the rat, a species with a larger body size than the mouse that allows multiple samplings, presents a better orthology with human immune cell markers and has drug pharmacokinetic profiles that are closer to those of humans. The experimental model used, which was established in an immunocompetent inbred strain known for its stable genetic background, closely mimics the worst situation faced in patients. The information collected using this experimental approach demonstrates to some extent that the two challenges defined above were partially reached. In addition, this represents a good basis both for future optimization of treatment procedures with this molecule and its derivatives and for investigations of modification of the status of macrophages and CD8+ T cells induced by the treatment.

## RESULTS

### Characterization of the M5-T1 rat mesothelioma cell line *in vitro*

**Figure 1:**
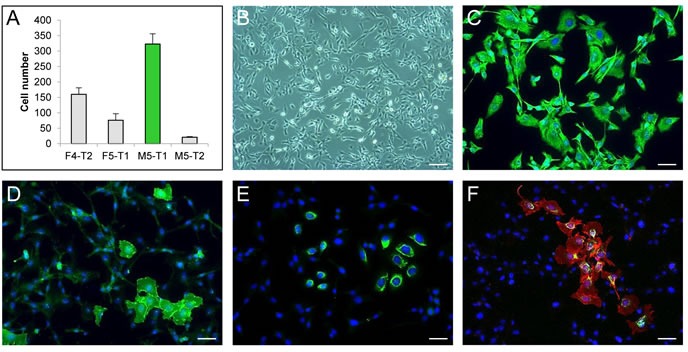
Characterization of the M5-T1 rat mesothelioma cell line *in vitro* **A.**, Invasive properties of the four rat mesothelioma cell lines tested in a Matrigel modified Boyden chamber assay. **B.**, Phase-contrast photomicrograph showing the morphology of M5-T1 cells. Scale bar, 106 μm. Simple immunostaining (green) of vimentin **C.**, E-cadherin **D.** and cytokeratin **E.** and double immunostaining of E-cadherin (red) and cytokeratin (green) **F.** of M5-T1 cells grown on glass coverslips. Nuclei were counterstained with DAPI (blue). Scale bars, 37 μm.

From the four malignant mesothelioma cell lines in our biocollection, which were isolated from F344 rats induced with asbestos, the M5-T1 cell line was selected for this study because of its high invasive capacity *in vitro* (Figure 1A). M5-T1 cells displayed a spindle-shaped morphology (Figure 1B). In addition, they all expressed vimentin but only a few of the cells retained the epithelial differentiation markers, E-cadherin and cytokeratin (0.76% ± 0.12% of the total population), as shown by immunofluorescence (Figures 1C-1F). These features are representative of a highly aggressive sarcomatoid phenotype.

### Optimal concentration/time combination allows curcumin to kill all tumor cells *in vitro*

We previously demonstrated that after 24 h treatment *in vitro*, 50% of M5-T1 cells remained viable in the presence of the highest concentration of cisplatin in the medium, 10.7 μM, while the highest concentration of curcumin, 100 μM, killed all tumor cells [29]. In addition, even after 48 h cisplatin treatment, resistant tumor cells were observed [29]. To extend these data, in this study we determined the curcumin concentration/time combination that led to the killing of all tumor cells *in vitro* without the generation of resistant cells, and these results were compared with those observed after treatment with 10 μM SAHA or 10 μM cisplatin. In addition, under similar treatment conditions, the cytotoxic action of the tested drugs was evaluated in parallel on a subnormal, non-tumorigenic mesothelial rat cell line, F1-0e. Within our biocollection, this cell line was selected as a reference, given its typical epithelioid cobblestone morphology comparable to normal mesothelial cells, and its maximal expression of epithelial markers (*Pdpn*, *Ezr*, *Msln* and *Cdh1*) and minimal expression of mesenchymal markers(*Vim*, *Acta2* and *Tgfb1*) [30].

**Figure 2:**
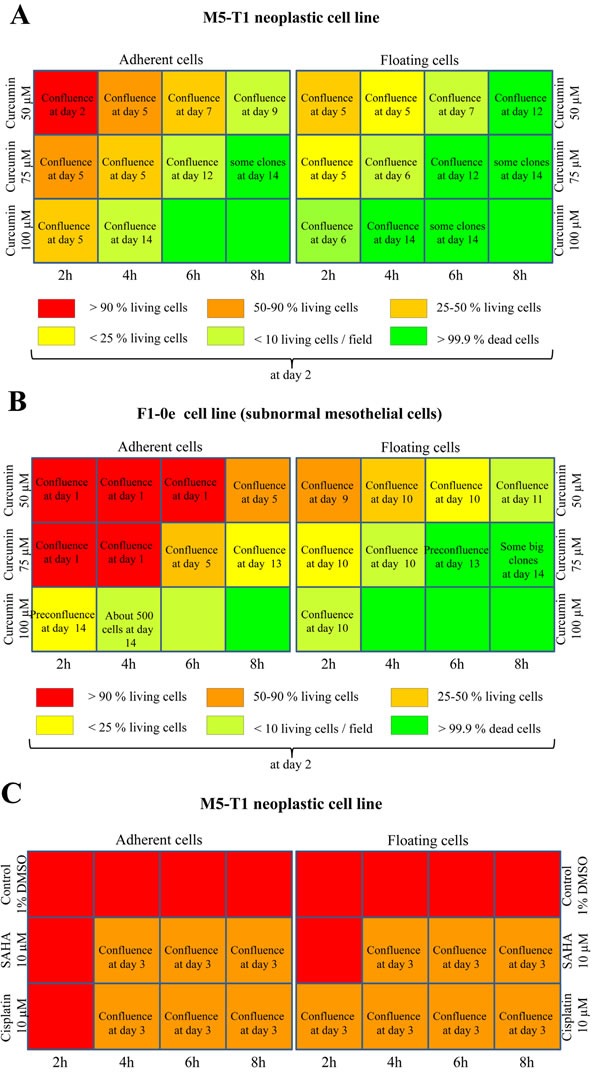

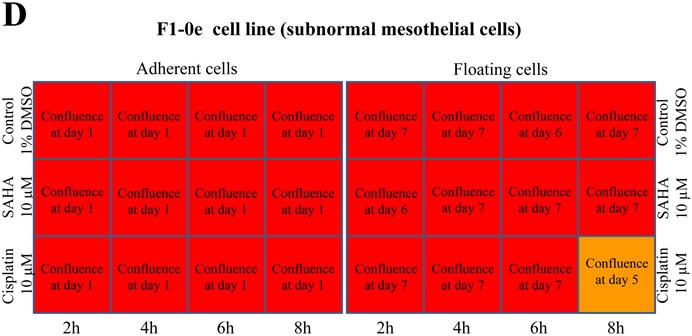
Concentration and time dependency of tumor-cell killing *in vitro* Scheme of the 12-well plates with a summary of the efficiency of treatment of M5-T1 tumor cells **A.**, **C.** with curcumin, and comparison with the results obtained on the reference non-tumorigenic subnormal F1-0e cell line **B.**, **D.**, representative example of three independent experiments. A color code represents the percentage of living cells observed 2 days after the end of treatment. Images of M5-T1 tumor cells and F1-0e cells for each treatment condition are provided in Supplemental Figures S1, S2 and S3. When the presence of resistant cells was observed after treatment, the number of days at which full confluence was observed is indicated in the corresponding well.

Overall, M5-T1 tumor cells differed from F1-0ep cells by a higher sensitivity to curcumin cytotoxicity. After incubation with 50 μM curcumin, a progressive decrease in the proportion of adherent living M5-T1 cells was observed from 2 h to 6 h incubation (Figure 2A and Supplemental Figure S1-A). This phenomenon was amplified and observed for shorter times of incubation with 75 μm curcumin (Figure 2A and Supplemental Figure S1-C), while 100 μM curcumin killed almost all tumor cells after 4 h treatment. In contrast, confluent F1-0ep cells were still observed after 2 h to 6 h incubation with 50 μM curcumin (Figure 2B and Supplemental Figure S1-B), and after 2 h and 4 h incubation with 75 μM curcumin (Figure 2B and Supplemental Figure S1-D). For floating cells collected at day 1, a significant proportion of living cells was still observed after treatment with 50 μM curcumin for 4 h and with 75 μM curcumin for 2 h (Figure 2A and Supplemental Figure S2C). However, contrary to the situation observed with the F1-0ep cell line, where almost all floating cells rapidly resumed their normal morphologies (Figure 2B and Supplemental Figure S2B), a high proportion of M5-T1 tumor cells remained affected by the treatment (Figure 2A and Supplemental Figure S2A and 2C). In contrast to treatments with curcumin, after treatment with 10 μM SAHA or 10 μM cisplatin, the decrease in density of cells/proportion of living cells in comparison with control wells was modest (Figures 2C, and 2D, and Supplemental Figure S3). Given these observations and the dramatic clinical signs reported in the toxicological evaluation of rats administered cisplatin i.p., including 23% loss of initial body weight at day 6 [31], for the *in vivo* experiments we decided not to include a group of tumor-bearing rats treated with cisplatin in this study. This decision was consistent with the recommendations on the welfare and use of animals in cancer research [21].

### Evidence of rapid killing of M5-T1 cells by curcumin *in vitro*, in contrast to SAHA

**Figure 3:**
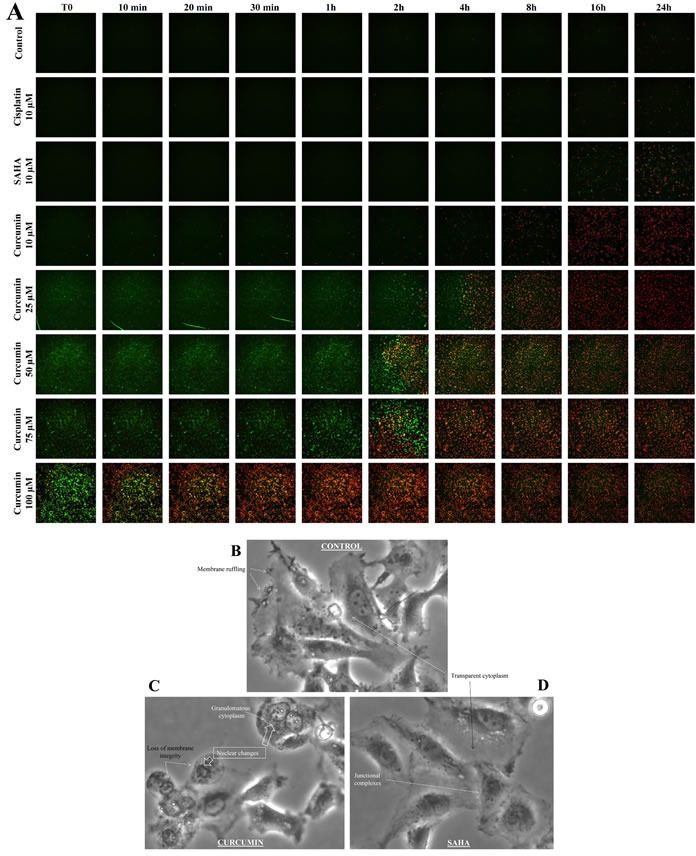
Timelapse fluorescence videomicroscopy of M5-T1 cells after treatment with cisplatin, SAHA or curcumin *in vitro* **A.**, M5-T1 cells were imaged at T0, 10 min, 30 min, 1 h, 2 h, 4 h, 8 h, 16 h and 24 h after treatment with 10 μM cisplatin, 10 μM SAHA, 10 μM, 25 μM, 50 μM, 75 μM or 100 μM curcumin in comparison with control cells (incubation with normal medium containing 1% DMSO). The green fluorescent YO-PRO-1 dye and the red fluorescent propidium iodide dye were used to detect the presence of apoptotic and necrotic cells, respectively. **B.**-**D.**, Morphological changes of M5-T1 cells after 2 h treatment with 100 μM curcumin in culture followed by incubation with normal medium for 3 h (C). Comparison with control cells (B) or cells treated with 10 μM SAHA (D). Changes included loss of membrane integrity and protrusion of cytoplasmic material, chromatin condensation and fragmentation, and granulomatous aspect of the cytoplasm. In contrast, junctional complexes, membrane ruffling and refringent inclusions within the cytoplasm were characteristic of living cells.

Using fluorescent markers of apoptosis and necrosis (YO-PRO-1 and propidium iodide, respectively) and time-lapse videomicroscopy, we observed that in comparison with other drugs, curcumin induces very early, in a dose-dependent manner, apoptosis followed by necrosis in M5-T1 cells (Figure 3A). Morphological observations of tumor cells in culture after only 2 h treatment with 100 μm curcumin followed by 3 h incubation with normal medium revealed that a number of the cells exhibited characteristic features of dead cells (Figure 3C) which were not seen with control tumor cells (Figure 3B) or those treated with SAHA (Figure 3D). These characteristics included loss of membrane integrity and protrusion of cytoplasmic material, and modification of the nucleus and cytoplasmic aspect, the latter losing its transparency and becoming granulomatous.

### M5-T1 mesothelioma presents invasive properties and a high mitotic index *in vivo*

**Figure 4:**
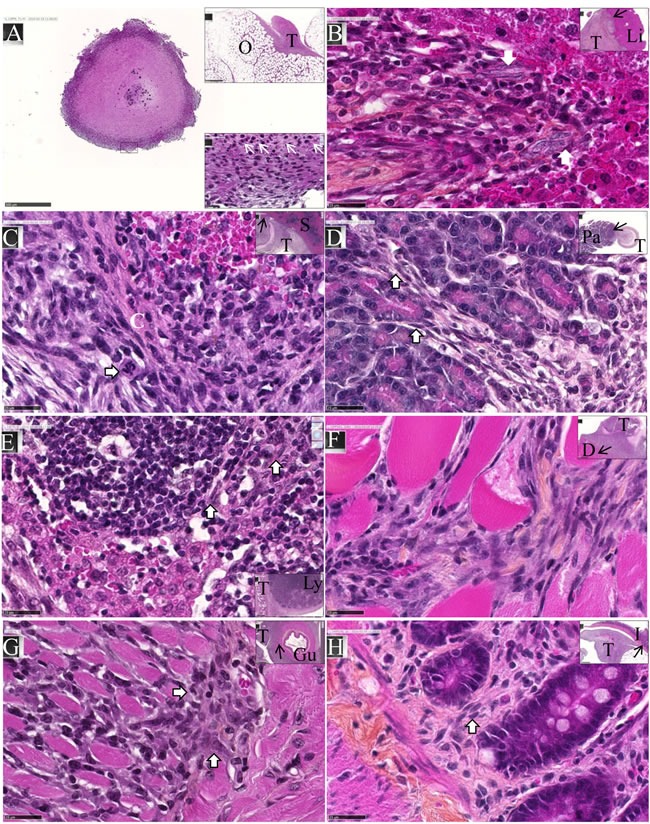
Experiment E1 M5-T1 cells exhibit invasive capacities *in vivo*. HPS staining, x800, the scale bars represent 25 μm. Inserts show general views, arrows indicate the localization of magnifications, scale bars represent 500 μm. **A.**, Example of one metastatic nodule from the peritoneal cavity, x50. The top insert shows tumor development on the omentum (O) with the formation of one of these nodules. The bottom insert exhibits a high magnification view (x800) of the area indicated by a black rectangle, with the presence of numerous monocytes/macrophages (white arrows). **B.**, Liver metastasis (T: tumor, Li: liver). The two white arrows in the magnification show the presence of elongated tumor cells with a large nucleus moving towards the liver parenchyma. **C.**, Spleen metastasis (S). The white arrow on the enlargement shows a mitotic figure with surrounding tumor cells invading the red pulp of the spleen after rupture of the capsule (C, in white). **D.**, Pancreatic metastasis (Pa). The two white arrows in the magnification show the presence of elongated tumor cells moving towards the mesenchymal space separating pancreatic acini. **E.**, Invasion of a mesenteric lymph node (Ly). Elongated tumor cells (white arrows) coming from the tumor growing in the mesentery are observed between cortical nodules close to lymphocytes. **F.**, Invasion of the diaphragm (D), showing both clusters and elongated isolated tumor cells moving between muscle fibers. **G.**, Invasion of the gut (Gu). The two white arrows in the magnification show the presence of tumor cells infiltrated between the longitudinal muscle cells and starting to invade the circular muscle cells of the muscularis externa. **H.**, Metastatic tumor nodule attached to the intestine **I.** The white arrow indicates the presence of elongated tumor cells invading the lamina propria of the mucosa. On the left part of the figure, tumor cells are also present within the outer longitudinal layer of the muscularis mucosae.

**Figure 5:**
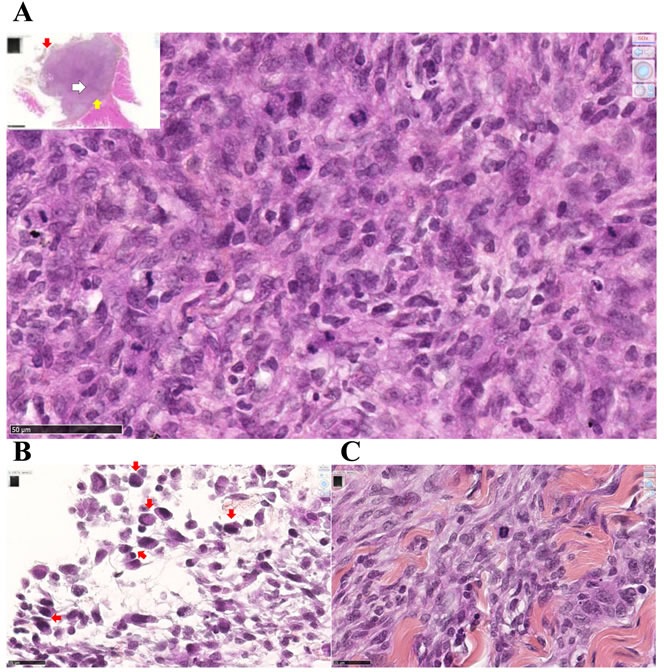
Experiment E1 Metastatic M5-T1 tumor tissue presents a high mitotic index. Histological aspects of a representative example of metastatic mesothelioma tissue invading the parietal peritoneum, HPS staining. (A), This image corresponds to areas with the highest cell density and mitotic index observed within the tumor tissue, x600. The insert shows a general view of the tumor tissue attached to the peritoneum, the position of this area being indicated with a white arrow, x50. (B), Peritoneal cavity side, the external layer of the tumor (position indicated with the red arrow in the insert in (A)) is characterized by the presence of macrophages (red arrows), x800. (C), On the parietal side (position indicated with the yellow arrow in the insert in (A)), the interface between the tumor and the invaded peritoneum is characterized by extended areas of fibrosis, x800.

After intraperitoneal inoculation of 5 × 10^6^ cells, tumor cells progressively occupied all the space previously covered by adipocytes in the normal omentum, producing an omental cake three weeks after tumor challenge. In parallel, numerous metastatic nodules of 1-2 mm diameter were observed in the peritoneal cavity (Figure 4A). Some of these nodules were found attached to different normal tissues, demonstrating the invasive capacities of tumor cells *in vivo* in the liver (Figure 4B), spleen (Figure 4C) and pancreas (Figure 4D). Evidence of tumor cells invading mesenteric lymph nodes (Figure 4E), the diaphragm (Figure 4F), muscularis externa of the gut (Figure 4G) and lamina propria of the colonic mucosa (Figure 4H) was also observed. The metastatic tumor tissue attached to the parietal peritoneum was frequently characterized by a high mitotic index, with 10 mitotic figures per x400 field (Figure 5A), the presence of macrophages in the external layer of the tumor (Figure 5B) and signs of fibrosis at the interface with the normal tissues (Figure 5C). Consideration of all these observations led to the conclusion that the M5-T1 tumor corresponds to a TNM grading of T4N1M1.

### Treatment effects of SAHA or curcumin *in vivo* differ by the extent of tumor necrotic areas

**Figure 6:**
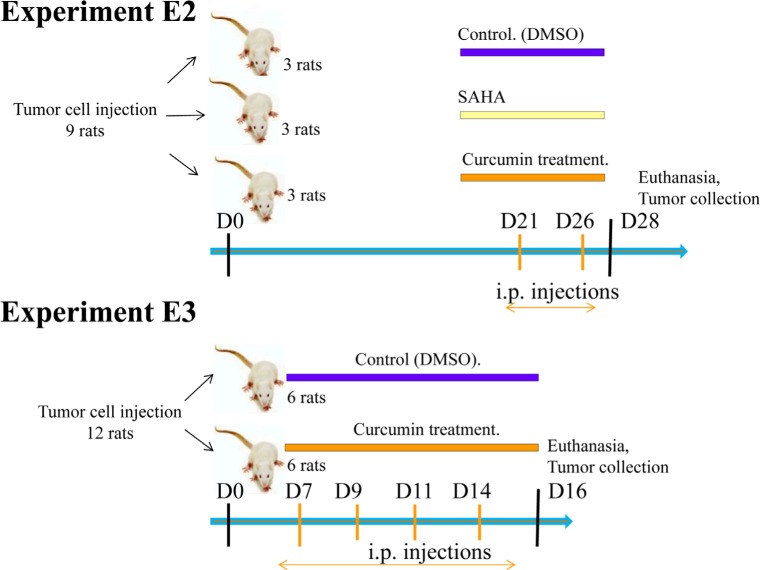
Scheme of the treatment procedures used for *in vivo* experiments

**Figure 7:**
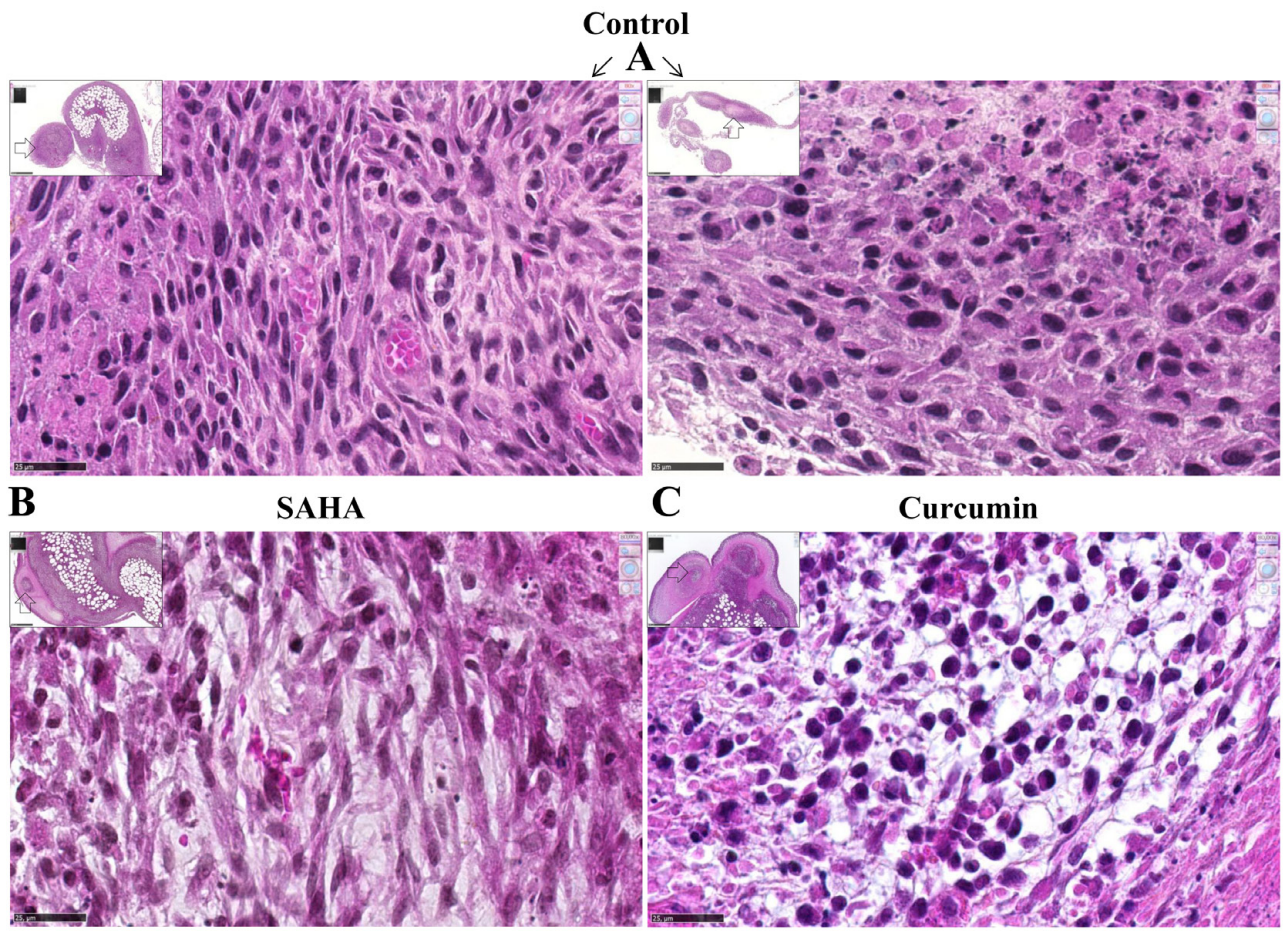

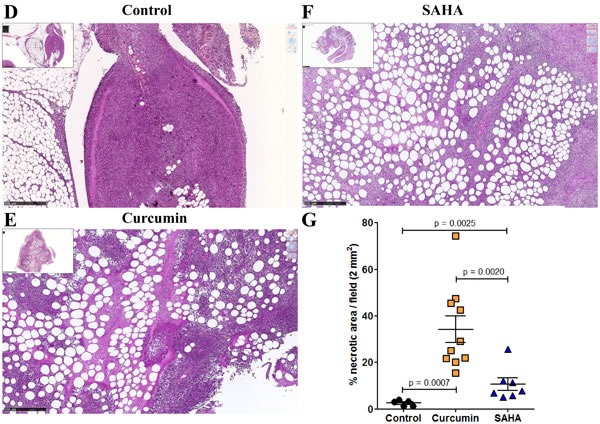
Images of the omental M5-T1 tumor at day 28, HPS staining (experiment E2) **A.**, Control tumor-bearing rats. These two examples show nodules released from solid tumors growing on the omentum, which contain small areas of necrosis either at the peripheral (left) or central (right) parts, x800, the scale bars represent 25 μm. Inserts show the positions of high magnification (white arrows), x50, the scale bars represent 500 μm. **B.**-**C.**, Treated tumor-bearing rats, central part of a nodule within the solid tumor growing on the omentum after two i.p. injections of SAHA (B) or curcumin (C) at days 21 and 26, x800, the scale bars represent 25 μm. Inserts show the position (white arrows) of the nodules surrounded by necrosis at the external parts of the tumors, x50, the scale bars represent 500 μm. **D.**-**F.**, Necrotic areas within the omental tumors, x50, the scale bars represent 500 μm. (D), Control tumor-bearing rat. (E), Tumor-bearing rat treated with curcumin. (F), Tumor-bearing rat treated with SAHA. Inserts show general views of the tumors (scale bars represent 2.5 mm). **G.**, Quantification of surfaces corresponding to necrotic areas.

Rats treated twice with 50 mg/kg SAHA or 750 μg/kg curcumin, at days 21 and 26 after tumor challenge (Experiment E2, Figure 6), presented no macroscopic evidence of tumor mass reduction. However, rats treated with curcumin were characterized by the yellowish color of the solid tumors, some parts presenting a softer surface compared with the dense tumors observed in untreated (control) rats. Histological analysis of the solid tumors collected from rats treated with curcumin (Figure 7C) showed large areas of necrosis (insert), the central part of the remaining nodules being characterized by a low tumor cell density, without cytoplasmic staining, and with signs of hemorrhage and a high density of macrophages. In contrast, comparable areas of the omental tumor from control rats or rats treated with SAHA presented high tumor cell density, homogeneous cytoplasmic staining and intact microvessels (Figures 7A and 7B). Quantification of the surfaces covered by necrotic cells demonstrated that the extension of necrosis within the invaded omentum was significantly higher after treatment with curcumin (Figures 7E and 7G) compared with treatment with SAHA (Figures 7F and 7G) and with control rats (Figure 7D).

### Treatment with SAHA or curcumin differ by the localization/density of immune cells

**Figure 8:**
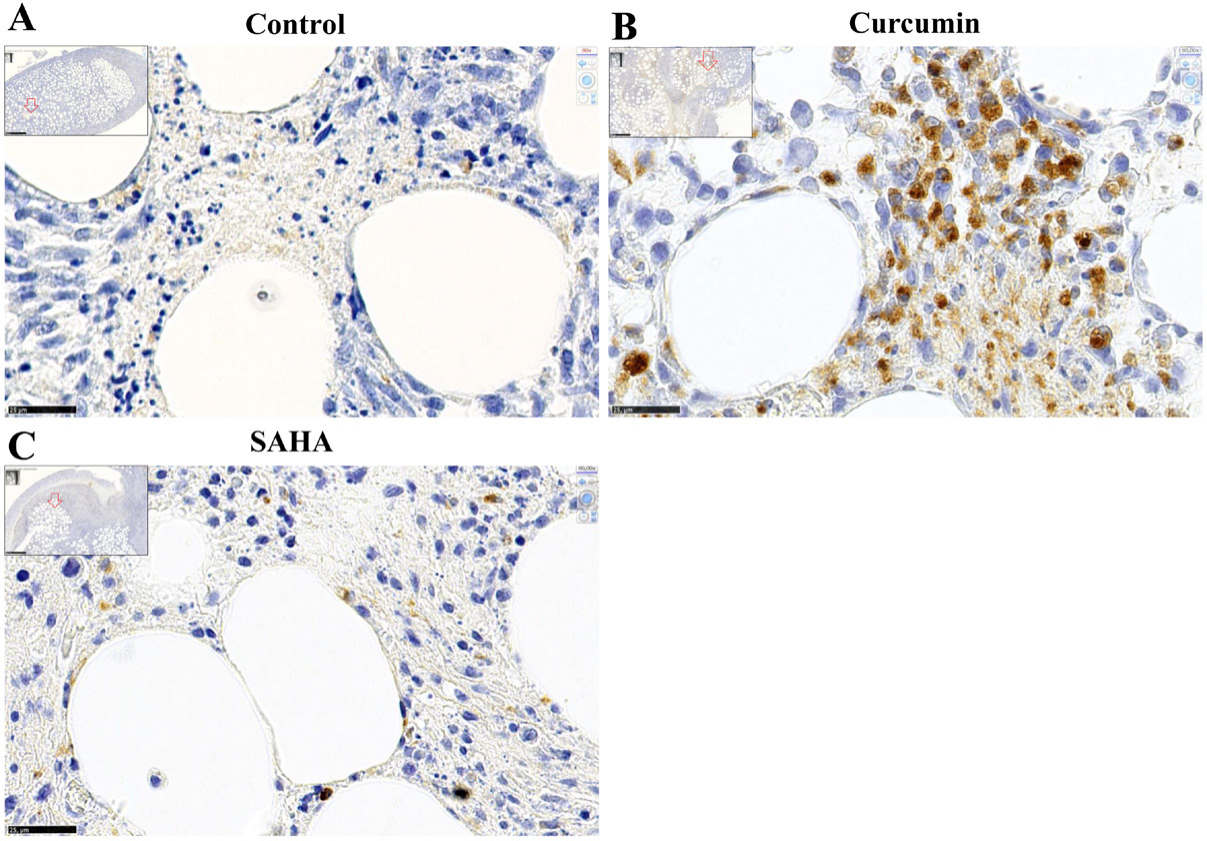

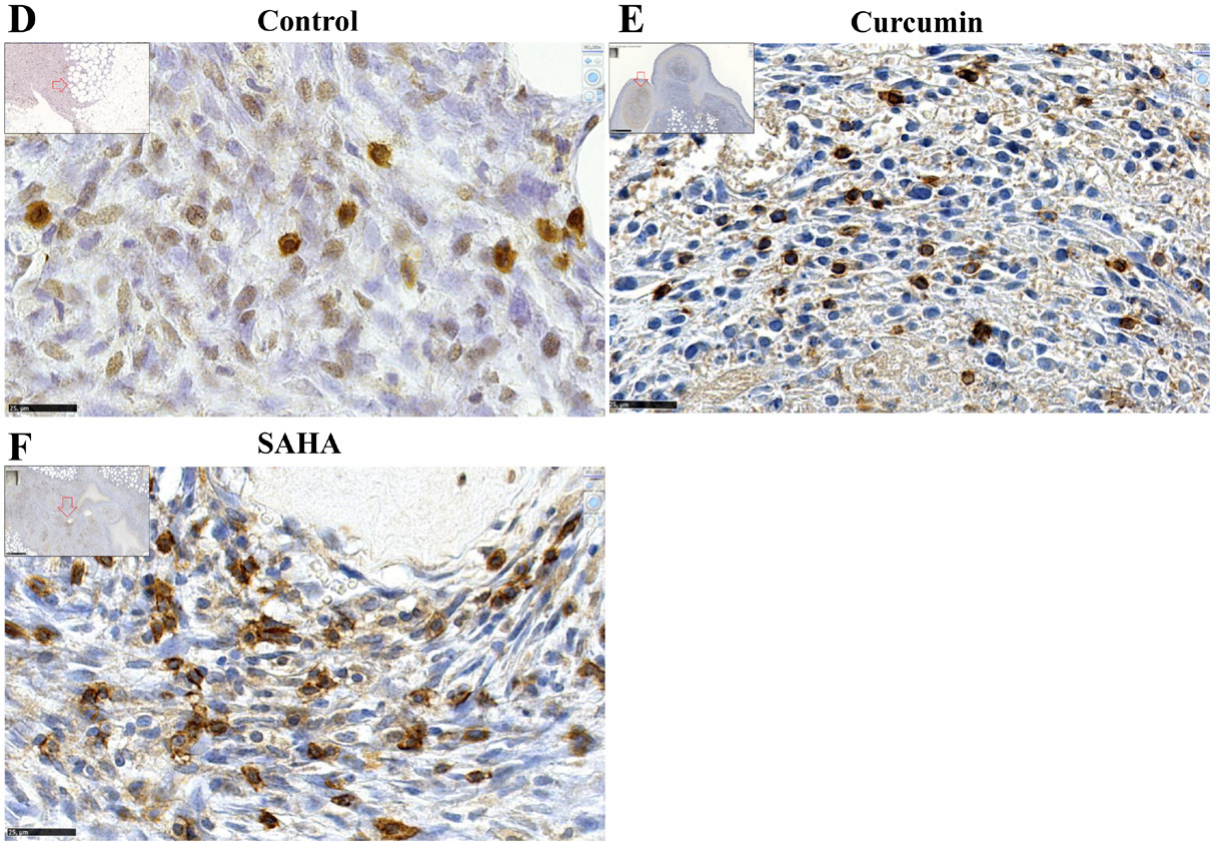

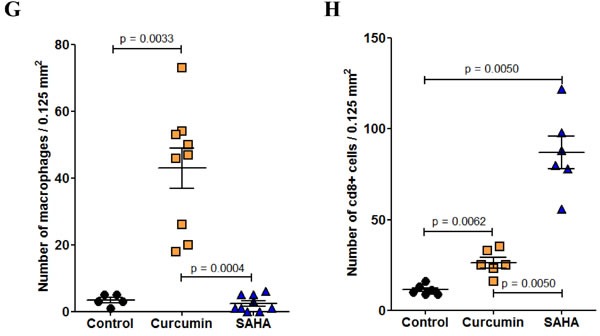
Immune cells infiltrating the omental tumor, day 28 (Experiment E2) **A.**-**C.**, Monocytes/macrophages, immunohistochemical staining with monoclonal antibody against the panmacrophage marker, ED1. (A), control rat, (B), rat treated with curcumin, (C), rat treated with SAHA, x800, the scale bars represent 25 μm. Inserts show larger views with open red arrows indicating the positions of photomicrographs in (A), (B) and (C), x50, scale bars represent 500 μm. **D.**-**F.**, CD8+ cells, immunohistochemical staining with monoclonal antibody against CD8+. (D), control rat, (E), rat treated with curcumin, CD8+ cells were observed mainly at the interface between necrotic areas and living tumor cells. (F), rat treated with SAHA, x800, the scale bars represent 25 μm. Inserts show larger views with open red arrows indicating the positions of photomicrographs in (D), (E), and (F), x50, scale bars represent 500 μm. Quantification of the number of monocytes/macrophages **G.** and CD8+ cells **H.** per field.

Untreated (control) rats were characterized by the rare presence of cells in the small necrotic areas within the omental tissue where the tumor initially grew (Figure 8A and insert). Globally, the tumor tissue of rats treated with SAHA contained few monocytes/macrophages, in particular in areas of necrosis within the invaded omental tissue where only isolated positive cells were found, as in control rats (Figure 8C and insert). Conversely, within the tumor tissue of the rats treated with curcumin, numerous monocytes/macrophages were concentrated at the interface between the necrotic areas and viable tumor cells within the invaded omental tissue (Figure 8B and insert).

In untreated (control) rats, isolated CD8+ cells were mostly present at the tumor front within the invaded omental tissue (Figure 8D). CD8+ cells were also present in the tumor tissues of rats treated with SAHA or curcumin, however their distribution/density differed in the two cases. In curcumin-treated rats, the CD8+ cells were distributed everywhere, including at the periphery of necrotic areas, close to dying cells (Figure 8E). In contrast, in SAHA-treated rats, the distribution was heterogeneous, with few CD8+ cells at the periphery of necrotic areas and a maximum density at different points of the unaltered tumor tissue, sometimes at the vicinity of growing nodules (Figure 8F). Quantification of immune cells revealed that curcumin-treated rats differed significantly from control rats or SAHA-treated rats, with a much higher density of monocytes/macrophages per field (Figure 8G). Additionally, the density of CD8+ cells per field in the tumor was significantly higher in SAHA-treated rats compared with curcumin-treated rats or control rats (Figure 8H).

### Multiple curcumin treatment reduces tumor mass and generates clusters of CD8+ T cells

**Figure 9:**
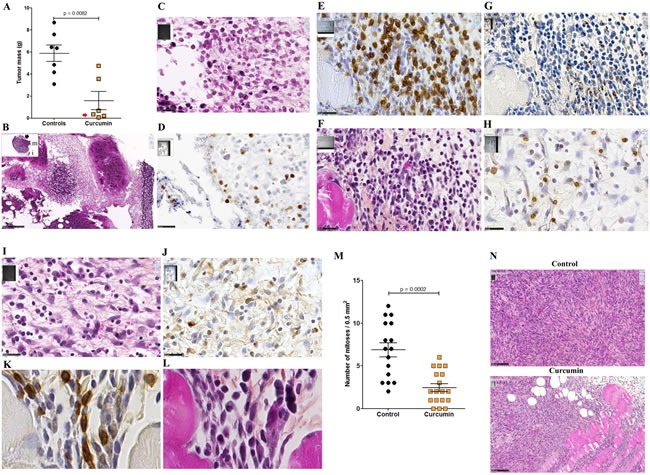
Reduction of tumor mass and presence of clusters of CD8+ T cells associated with residual tumors (Experiment E3) **A.**, Decrease of the total tumor mass at day 16 compared with untreated rats (controls). The red arrow indicates the subgroup of rats presenting only small residual tumor masses in the peritoneal cavity. **B.**, Representative example of one of these small residual tumor masses in this subgroup of rats treated with curcumin. Insert, general view, x13.6, the scale bar represents 2.5 mm, the residual tumor developed on the mesentery, “m” and “i”, indicate muscularis and intestinal mucosa, respectively. Large view: area indicated by the black arrow in the insert showing large fields of necrotic tumor cells, x100, the scale bar represents 250 μm. **C.**, Magnification of the area indicated by the black rectangle in (B), showing the external part of a small islet of residual tumor cells surrounded by necrotic cells and infiltrated by numerous monocytes/macrophages, x800, the scale bar represents 25 μm. **D.** Immunohistochemical staining of the same area shown in (C) with the monoclonal antibody against the panmacrophage marker, ED1, showing monocytes/macrophages moving into a large field of necrotic tumor cells. **E.**-**G.**, Analyses of immune cells infiltrating the external part of a residual tumor, subgroup of rats treated with curcumin, x800, the scale bars represent 25 μm, immunohistochemical staining with anti-CD8 (E) and anti-ED1 (G) monoclonal antibodies, and HPS staining (F). **H.**-**J.**, Analyses of immune cells infiltrating the external part of a metastatic tumor, group of control rats, x800, the scale bars represent 25 μm, immunohistochemical staining with anti-CD8 (H) and anti-ED1 **J.** monoclonal antibodies, and HPS staining **I.** **K.**, **L.**, High magnifications of CD8+ T lymphocytes infiltrating the internal part of the residual tumor shown in (E-G), immunohistochemical staining with anti-CD8 (K) and HPS staining (L), x3800. **M.**, Quantification of the number of mitoses per field in the residual tumor shown in (E-G and K,L), and comparison with the metastatic tumor from an untreated (control) rat shown in (H-J). **N.**, Two representative fields used for the quantification, x200, the scale bars represent 100 μm.

**Figure 10:**
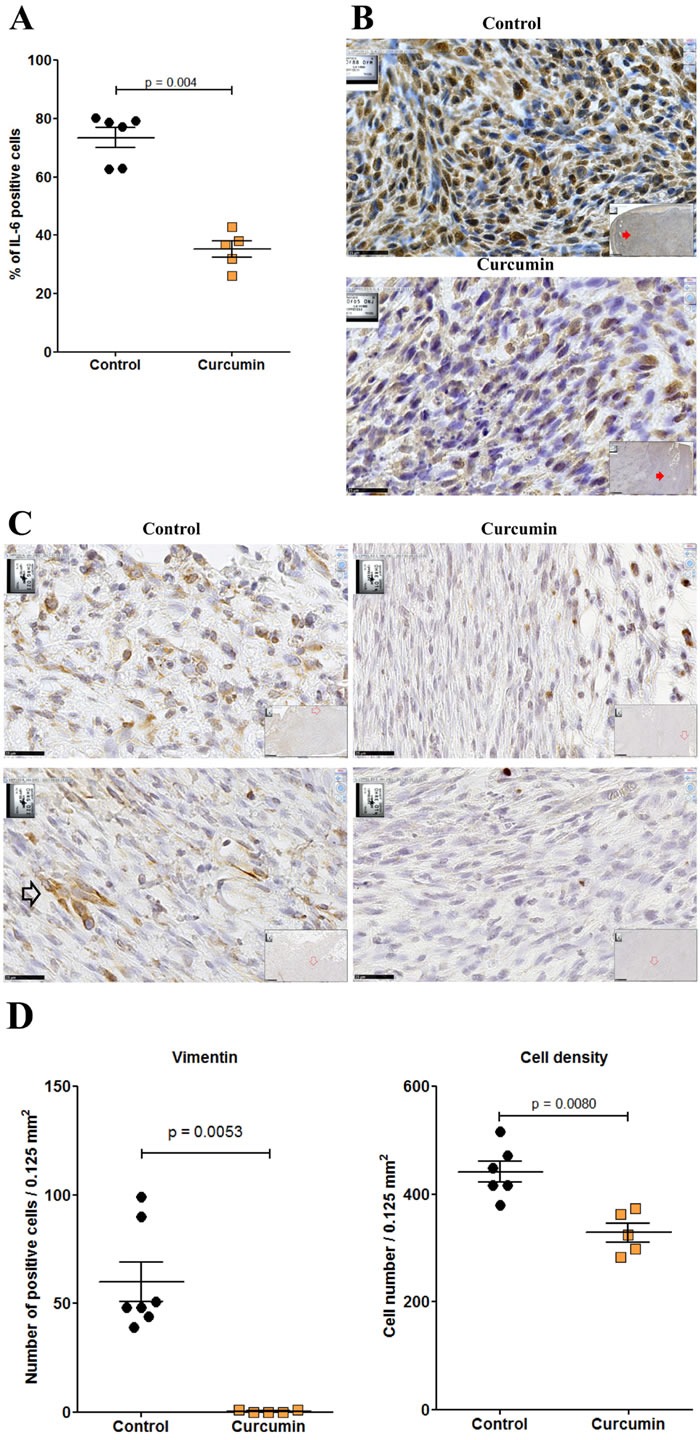
Residual tumor of rats treated with curcumin exhibited a significant decrease of IL-6 and vimentin expression **A.**, Quantification of the numbers of IL-6 positive cells per high power field. **B.**, Immunohistochemical staining of metastatic tumor (control rat, top) and residual tumor (subgroup of rats treated with curcumin, bottom) with anti-IL6 monoclonal antibody. Large views, x 800, the scale bars represent 25 μm. Inserts show general views (x 50), red arrows indicating the localization of magnifications, scale bars represent 500 μm. **C.**, Two representative high-magnification views (x800, the scale bars represent 25 μm) of tumor cells after immunohistochemical staining of a metastatic tumor (control rat, left) and a residual tumor (subgroup of rats treated with curcumin, right) with anti-vimentin monoclonal antibody. Top, external parts of the tumors. Bottom, central parts of the tumors. The open black arrow (control rat) illustrates the typical morphology and dense staining observed with some tumor cells. The only strongly positive cells observed in the sections from treated rats correspond to lymphocytes and macrophages. **D.**, Quantification of the numbers of vimentin positive cells per high power field (left) and of cell density (right).

Given the higher extent of necrotic areas and the absence of foci of resistant tumor cells observed within the tumor tissue after treatment with curcumin, in comparison with SAHA (in experiment E2 described above), experiment E3 focused on the comparison of multiple curcumin treatments vs untreated rats (Figure 6). Rats given four injections of 1.5 mg/kg curcumin at days 7, 9, 11 and 14 after tumor challenge presented a significant reduction in their mean total tumor mass compared with controls. This result was particularly marked in a subgroup of four of the six rats (Figure 9A, red arrow) presenting no metastases in the liver, pancreas and diaphragm and only small residual tumor masses in the submucosa of the gut and/or parietal peritoneum. These residual tumors were characterized by the presence of extended necrotic areas at their periphery (Figures 9B and 9C) and the presence of numerous monocytes/macrophages (Figure 9D). In this subgroup, an important concentration of clusters of large CD8+ T lymphocytes was also observed at the periphery of residual tumor tissue (Figures 9E and 9F). In addition, immunohistochemical staining of the same area revealed that the distribution and morphology of the ED1+ cells did not correspond, the presence of monocytes/macrophages being limited and localized (Figure 9G). The comparison of these features with observations made on metastatic tumors from the group of untreated (control) rats revealed that a few isolated CD8+ T lymphocytes were present (Figure 9H and 9I) as well as some monocytes/macrophages (Figure 9J). In addition, examination of high-magnification images of CD8+ T lymphocytes infiltrating the inner part of residual tumors in treated rats showed moving cells following each other and presenting a fusiform morphology, in contrast to spherically shaped CD8+ T lymphocytes observed in untreated (control) rats (Figures 9K and 9L). Residual tumor tissue also presented a significantly reduced number of mitoses per field in the areas of compact tumor growth compared with comparable areas in tumors from untreated (control) rats (Figures 9M and 9N). Finally, multiple curcumin treatments produced a pronounced decrease both in the proportion of positive cells (Figure 10A) and intensity of staining (Figure 10B) after immunohistochemical staining with an anti-IL6 monoclonal antibody, suggesting a change in tumor microenvironment. This change was further evidenced by the disappearance of the mesenchymal character of this tumor, a characteristic observed previously [30], revealed by a dramatic decrease in the expression of vimentin in all parts of the tumor tissue (Figures 10C and 10D), in parallel with a decrease in cell density (Figure 10D).

### Normal tissues without metastases present evidence of increased numbers of circulating CD8+ T cells

In normal rats, the presence of T lymphocytes in the pancreas, and in particular of CD8+ T cells, is extremely rare. Conversely, some areas within the pancreas of rats without metastases from the subgroup treated with curcumin (red arrow in Figure 9A) were characterized by the presence of numerous cells densely stained with a very high nucleus to cytoplasm ratio. Further immunohistochemical staining of these areas with anti-CD3 and anti-CD8 monoclonal antibodies revealed that they corresponded to T lymphocytes, and particularly CD8+ T lymphocytes (Supplemental Figure S4A), some of them showing the same morphology as in Figure 9K. In contrast, the pancreas of control (untreated) rats was characterized by the presence of numerous circulating tumor cells, while CD3+ and CD8+ lymphocytes were absent (Supplemental Figure S4B).

Like the pancreas, the liver of treated rats presented no metastatic nodules attached to the capsule. Rather, the surface of the liver revealed a thickening of the capsule and the presence of CD8+ T cells (Supplemental Figure S5A, top), which contrasted strikingly with the dramatic invasion of the liver surface and absence of CD8+ T cells observed in control (untreated) rats (Supplemental Figure S5B, top). Within the liver parenchyma of treated rats, the vicinity of venules was also characterized by numerous CD8+ T cells, revealing their mobility, most of them in contact with minute tumor cells (Supplemental Figure S5A, bottom), conversely with the situation found in control (untreated) rats (Supplemental Figure S5B, bottom). Quantification of CD8+ T cells in these areas also revealed a significantly higher CD8+ T cell to tumor cell ratio in treated rats compared with control rats (Supplemental Figure S5C).

### Induction of a cell-mediated immune response in the spleen, and small tumor cell foci

This presence of circulating CD8+ T cells in normal tissues of curcumin-treated rats was corroborated by observations made in the white pulp of the spleen. In treated rats, the lymphoid sheath surrounding the central artery of the splenic nodules was characterized by larger, intensely stained and densely packed lymphocytes, which corresponded to CD8+ T cells after immunohistochemical staining (Supplemental Figure S6A), in contrast with control (untreated) rats. Numerous CD8+ T cells were also observed in the red pulp, some of them grouped (Supplemental Figure S6B).

**Figure 11:**
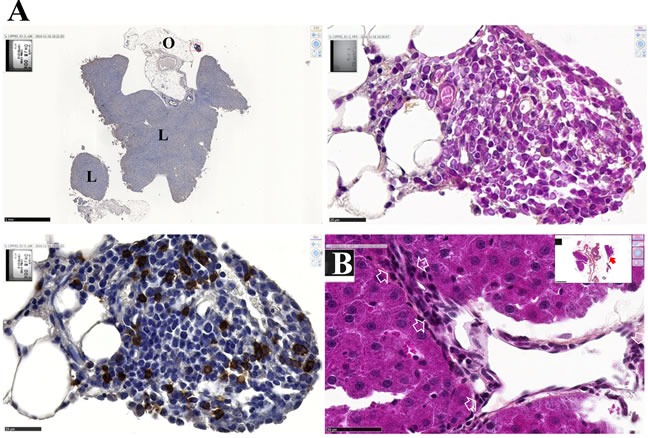
Morphological changes of residual tumor cells in contact with activated lymphocytes **A.**, Images of a minute cluster of residual tumor cells remaining in the omentum (O) and vicinal to the liver (L) after immunohistochemical staining with anti-CD8 monoclonal antibody. Top left, general view, x2.5, the scale bar represents 1 mm. Bottom left, enlargement of the field denoted by the red rectangle (x800, the scale bar represents 25 μm), and corresponding HPS staining showing morphological changes of tumor cells (top right). **B.**, Battlefield at the surface of the liver. General view from another section of the same rat as in (A), (insert, x2.5 the scale bar represents 1 mm), and detailed view of the field denoted by the red arrow (x600, the scale bar represents 50 μm) showing numerous activated lymphocytes in contact with residual tumor cells and avoiding them to penetrate the liver parenchyma. Some of these tumor cells exhibit morphological changes (white arrows) as compared with images from control (untreated rats) as seen in Figure 4B and Supplemental Figure S5B.

In parallel, minute foci of tumor cells localized on the omentum in the vicinity of the liver were characterized by a high proportion of CD8+ T cells (Figure 11A). Lymphocytes found in these structures or present at the surface and inside the liver parenchyma both presented the same typical morphologies as those illustrated in Figures 9K-9L. This fact, and their accumulation in places where tumor cells attempt to penetrate normal tissues (Figure 11B), tends to attest to their functionality and high mobility. Tumor cells found in close contact with several of these lymphocytes also revealed changes in their normal morphologies (size/shape, aspects of the nucleus and cytoplasm staining) (Figure 11B) suggesting modification of their metabolic state and behavior.

## DISCUSSION

In this study, we report data and observations on a rat model of sarcomatoid MM that could open up new prospects for the treatment of this devastating disease, which is associated with cancer cell dissemination and poor outcome. In addition, this model could represent a good basis for evaluating the immune status modification induced by the treatment. The capacity of curcumin administered intraperitoneally to kill a significant proportion of cancer cells, to induce an influx of macrophages in areas of apoptotic cells, and finally to produce an accumulation of CD8+ T cells at the periphery of residual tumor tissue and an infiltration of normal tissues was demonstrated. In contrast, the cytotoxic properties of SAHA were hampered by the emergence of resistant tumor cells, both after *in vitro* and *in vivo* treatment.

Our results *in vitro* are in agreement with the observation that treatment with curcumin totally inhibited cell growth by mechanisms that are not cell-type dependent [32]. They also confirm results obtained previously on various cancer cell types demonstrating that this molecule acts in parallel on different cell signaling pathways [33, 34] through its ability to bind directly to a plethora of proteins [35]. We confirm our previous data [29] and show that an optimal combination of concentration and time leads to the death of all tumor cells, avoiding the emergence of resistant cells, in contrast to treatment with SAHA or cisplatin. This dose dependency and time dependency have been documented previously [36]. Our observation that at 100 μM curcumin, all mesothelioma cells are definitively killed is in good agreement with the work of Romero-Hernández and colleagues on human astrocytoma cell lines, showing that at a high concentration, curcumin-induced apoptosis involves caspase-independent processes [37]. Furthermore, we provide evidence that, in contrast to the non-tumorigenic reference cell line used, almost all M5-T1 tumor cells are definitively killed after 8 h exposure to 75 μM curcumin. This result is supported by the very quick lethal effect of curcumin at high dose observed by Clark and colleagues, who also mentioned that no additional effect was observed at 48 h and 72 h compared with 24 h [38]. Conversely, this result contrasts with reports on the growth-inhibitory effects of liposomal curcumin after 72 h [39], suggesting that for nanoformulations containing curcumin, the control of the rate of release of the drug is a critical parameter for its efficiency to kill tumor cells. Evidence for a rapid cytotoxic effect of curcumin has previously been reported by Killian and coauthors, showing that this molecule acts as an inhibitor of IκB kinase β as early as 2 h after treatment [40]. Our phase-contrast and time-lapse videomicroscopy images of apoptotic and necrotic tumor cells after 2 h treatment with curcumin are also consistent with the work of Kunwar and colleagues, showing very quick localization of the drug to the cell membrane and nucleus of MCF7 cells [41]. Finally, although the potential of curcumin against mesothelioma cells *in vitro* has already been demonstrated [25–28], the range of concentrations (10, 30 and 50 μM) and the single time (24 h) used led to a modest reduction in cell viability, including cell retraction and cell rounding without apoptosis induction, suggesting a mechanism of autophagy [26]. Interestingly, the results we obtained with 50 μM are in good agreement with these observations, demonstrating that at this concentration tumor cell growth quickly resumes after treatment, a condition that must be avoided for *in vivo* experiments.

In this study, M5-T1 cells that retained the epithelial differentiation markers E-cadherin and cytokeratin represented less than 1% of the population, suggesting that curcumin is very effective in killing tumor cells with a dedifferentiated sarcomatoid phenotype known to develop resistance to conventional and targeted cancer therapies [42]. *In vivo* , in contrast to SAHA, i.p. administration of curcumin did not generate foci of resistant cells in the solid tumor. This observation is consistent with the recent confirmation that, conversely to 5-FU and platinum derivatives, curcumin inhibits specific pathways leading to treatment resistance [43], and with the suggestion that this molecule is particularly well adapted to the treatment of aggressive and/or resistant tumor cells [44]. The disappearance of nodules in the residual solid tumors, as well as areas exhibiting a maximum density of mitoses at their periphery, in comparison with untreated rats, suggests a link with the potential of curcumin to target cancer stem cells. This has been reviewed recently [45]. Besides the significant reduction of the mean total tumor mass, another interesting point concerns the dramatic decrease in the extent of metastasis. The absence of metastatic tumor tissue observed at the surface of the spleen, liver, diaphragm and parietal peritoneum emphasizes the interest of the route of administration used in this study. Our results are also consistent with recent clinical studies showing that the treatment of colorectal peritoneal metastases with intraperitoneal chemotherapy, as compared with systemic chemotherapy, contributes to reducing tumor burden [46]. Among studies reporting the inhibitory effect of intravenous administration of liposomal curcumin on tumor xenografts in the athymic mouse, doses of 40 mg/kg [33] or 50 mg/kg [47, 48] were used, which corresponded to the maximum volume that could be injected. In our study, the final amount of curcumin tested for i.p. injection, 1.5 mg/kg, which produced a significant reduction in the mean tumor mass, was very low compared to the 25 mg/kg i.v. dose used in a control group by Alam *et al*. [49]. Given the very low systemic toxicity reported for DMSO in different species [50], and the modest volume (1/3 v/v) used in our study, there appears to be a considerable margin in the maximum tolerated dose of this compound that could be used i.p. for chemotherapy. For example, in rats, i.p. injections of 2 ml/kg (50% in saline) have even been shown to exhibit hepatoprotective properties [51].

Inflammation plays a considerable role in cancer, and this is particularly the case for MM. The use of phytochemicals to inhibit inflammatory processes implicated in cancer promotion has been suggested [52], a strategy that has been successful against the development of some aggressive types of cancer [53]. In this study, we confirm our previous suggestion that among phytochemicals, curcumin appears promising in limiting the wasting consequences of the progression of such aggressive cancers, as a suppressor of several proinflammatory factors secreted by the tumor [54]. Our results also tend to confirm the interest of the treatment strategy recently proposed by Benvenuto and colleagues, based on the administration of polyphenols in the serous cavity to reduce the chronic inflammatory response associated with MM development [55]. Curcumin has been recognized as an immunomodulatory agent that can modulate the activation of T cells, B cells, macrophages, neutrophils, NK cells and dendritic cells, through the downregulation of various proinflammatory cytokines [56]. In contrast with many anticancer drugs that exhibit deleterious effects on immune cells, prolonged i.p. curcumin injections do not impair the cytotoxic function of NK cells, while they do maintain the level of Th1 regulatory cytokines and enhance the antigen-induced proliferation potential of T cells [57]. Interestingly, curcuminoids also inhibit the induction of nitric oxide synthase in activated macrophages [58], an effect that was established *in vitro* in 1995 [59] and confirmed *in vivo* three years later [60]. The observation of an influx of macrophages towards areas of dead/apoptotic tumor cells within the tumor tissue is consistent with the finding that curcumin administration increased the amount of plaque-forming cells in the spleen after immunization with SRBC, enhanced bone marrow cellularity and increased the phagocytic activity of macrophages in Balb/c mice [61]. Finally, our results highlight the therapeutic utility of curcumin in stimulating the immune reaction of the host against the tumor, and tend to confirm the myelopotentiating effect of this molecule, which has been described in a mouse tumor model [62].

In this study, an important concentration of clusters of CD8+ T lymphocytes was observed at the periphery of small tumor masses that remained in the peritoneal cavity or infiltrating microscopic masses in the omentum vicinal to the liver, in contrast with the case of rats treated with SAHA that presented numerous foci of isolated cells in areas of compact tumor growth in the solid tumor. The latter case could be explained by an increased expression of cancer antigens induced by inhibitors of histone deacetylases (iHDACs), which has previously been documented both *in vitro* and *in vivo* [63]. SAHA-induced increases in the expression of MHC class I-related chain molecules A and B have also been observed recently in two hepatocellular carcinoma cell lines, resulting in an enhancement of immune recognition of tumors by innate immune cells [64]. Concerning the dense T-cell accumulation following treatment with curcumin, this has previously been observed in tumor-bearing mice, suggesting that the combination of multitargeting drugs with adoptive therapy could have the potential for clinical application [65]. Luo and coauthors also reported a proliferation of CD8+ T cells with enhancement of IFN-γ secretion and cytotoxicity specifically against 3LL tumor cells in tumor-bearing mice following i.p. treatment with curcumin [66]. Moreover, they documented the bimodal effect observed *in vitro* with this molecule, with a high dose of curcumin decreasing T-cell numbers whereas a low dose increased the T-cell number derived from 3LL tumor-bearing mice. In correlation with other previous observations made *in vivo* showing that low doses of curcumin downregulated the Bax level while augmenting Bcl-2 expression in CD4+/CD8+ T cells, thereby protecting the cells from tumor-induced apoptosis [67], our results also agree with the attenuation of tumor-induced suppression of cell-mediated immune responses described by Bhattacharyya and colleagues [68]. Finally, in contrast with previous reports mentioning the presence of CD8+ macrophages in the context of glioma or various other CNS pathologies [69], our observations, including HPS staining and immunohistochemical labeling with anti-CD8 and anti-ED1, show that both the distribution and the morphology of these clusters of CD8+ cells differ from those of monocytes/macrophages. Overall, these observations, together with the disappearance of monocytes/macrophages from areas of compact tumor growth in the residual tumor mass and a significant decrease in the mitotic index, suggest a profound modification of the immunosuppressive environment produced by the tumor. Further evidence for this modification is also provided by the observation of a significant decrease in IL6 and vimentin expression in residual tumors. This observation is well correlated with findings by So and colleagues who demonstrated that different types of gynecologic cancer cells exposed to IL6 following co-culture with mesenchymal stem cells acquired facilitated metastasis and invasion by promoting EMT [70]. To summarize, our findings tend to demonstrate that the marked decrease in proinflammatory cytokines such as IL6, associated with a loss of mesenchymal character, both produced by curcumin treatment in our aggressive model of MM, may facilitate the emergence of an immune response directed against tumor cells. This T-cell-mediated response is evidenced by an increased production of CD8+ T cells in the spleen and their numerous presence, sometimes in contact with isolated tumor cells, both in normal tissues preserved from metastases, and in minute foci of residual tumor cells vicinal to these normal tissues, where these latter present important changes of their morphology.

In conclusion, these data open up interesting new prospects for the therapy of malignant mesothelioma with curcumin, in particular the sarcomatoid subtype. Future studies will determine the best sequence/dose(s) of curcumin or its many derivatives to use to optimize the specific immune response directed against M5-T1 cells. A complementary approach would also be the combination of curcumin/derivatives with current chemotherapeutic agents to reduce the dose used and the associated systemic toxicity of the latter. Finally, the treatment of these tumor cells with SAHA represents an interesting investigative tool for basic research in oncoimmunology, in particular to improve our understanding of the process of infiltration of mesothelioma by CD8+ T lymphocytes *in vivo*.

## MATERIALS AND METHODS

### Rats and M5-T1 mesothelioma cell line

Fischer F344 rats were obtained from Charles River Laboratories (L’Arbresle, 69, France) and maintained under standard conditions in the UTE IRS-UN animal-holding area, in agreement with European Union guidelines for the care and use of laboratory animals in research protocols. The experiments were approved by the regional ethical committee for animal experiments (CEEA.2011.38, CEEA.2013.7, and project n° 01257.03). Rats were fed a pelleted standard diet (RM1, Special Diet Services, Witham, Essex, UK) with tap water *ad libitum*, anesthetized via an isoflurane chamber (Forene^®^, Abbott France) and euthanized with Dolethal^®^ (Centravet, Pluduno, Plancoët, France) for experiments E2-E3.

The M5-T1 mesothelioma cell line used in this study was selected from a biocollection established in 2011 [ https://migratech.inserm-transfert.fr/srv/tech/2/index100.asp?cl=CL] after 136 to 415 days of induction with 10 mg crocidolite fibers suspended in 0.5 ml 0.9% NaCl, administered intraperitoneally to rats (UICC analytical sample, ref. 02704A, Neyco, 75017 Paris, France). After 378 days of induction, one male rat was necropsied about one hour after death, presenting signs of hemorrhage, widespread neoplastic implants and nodules in the peritoneal cavity, as previously described [30], which, when dissociated with a scalpel and cultured, led to the M5-T1 cell line.

### Cell culture and chemicals

Cells were cultured in RPMI 1640 supplemented with 10% heat-inactivated fetal calf serum, 100 U/ml penicillin, 0.1 mg/ml streptomycin and 2 mM L-Glutamine (Sigma-Aldrich, St Louis, MO, USA), in a humidified atmosphere of 5% CO_2_ at 37°C. Stock solutions of curcumin (Sigma-Aldrich, L’isle d’Abeau Chesnes, 38, France), suberoylanilide hydroxamic acid (SAHA), (Toronto Research Chemicals Inc., Canada) and cisplatin (Mylan, St Priest, 69, France) were dissolved in DMSO and preserved at −20°C before use.

### Modified Boyden chamber invasion assay

The *in vitro* invasive properties of the four neoplastic cell lines (M5-T1, M5-T2, F4-T2 and F5-T1) were assessed using a modified Boyden chamber assay. An aliquot of 10^5^ cells in serum-free medium was placed in the upper compartment of the invasion chamber (BD BioCoat Matrigel Invasion Chamber, BD Biosciences, Bedford, MA, USA). The lower compartment was filled with RPMI containing 10% FCS. The chambers were incubated for 17 h at 37°C. The filters were then fixed in methanol and stained with hematoxylin. Quantification of the invasion assay was performed by counting the number of cells at the lower surface of the filters (23 fields at 400x magnification).

### Immunofluorescence

Subconfluent monolayers of M5-T1 cells cultured on glass coverslips were fixed with methanol. The coverslips were incubated with either a mouse monoclonal antibody to vimentin (clone V9; Dako, Glostrup, Denmark), a mouse monoclonal antibody to E-cadherin (clone 36, BD Transduction Laboratories, BD Biosciences, San Jose, CA) or a mouse monoclonal antibody to cytokeratin (clone AE1/AE3, Dako). Monolayers were next incubated with Alexa Fluor 488- or Alexa Fluor 594-coupled anti-mouse IgG (Molecular Probes, Eugene, OR, USA). Nuclei were stained with DAPI (Molecular Probes). The staining was analyzed using a Zeiss AxioImager fluorescence microscope (Carl Zeiss MicroImaging GmbH, Germany).

### Analysis of the concentration and time dependency of tumor cell killing by curcumin *in vitro*

Cell lines were seeded at 1 × 10^5^ cells/well at day -2, in 12-well plates (Nunclon delta, Nunc AS, Denmark). These conditions were selected in order to obtain a cell density in the central part of the wells corresponding to the beginning of three-dimensional growth of the M5-T1 cells (which have the propensity to produce spheroids [29, 30]). For growth-inhibition and cell-killing assays, 48 h after adhesion (day 0) the medium was replaced with fresh medium containing increasing concentrations of curcumin (50, 75 or 100 μM), 10 μM SAHA, 10 μM cisplatin or 1% DMSO (control wells). F1-0e and M5-T1 tumor cells were incubated for 2 h, 4 h, 6 h or 8 h in these conditions, after which the medium was replaced with normal medium for 24 h. At day 1, floating cells were resuspended after two flushes with the medium for each well, the cells were centrifuged, and the pellets were resuspended in fresh medium and placed into the wells of two new 12-well plates. In parallel, fresh medium was added to each well in the first two 12-well plates (adherent cells). At day 2, the central part of each well of both plates was photographed at x100 and x320 magnification using a Zeiss Observer.Z1 AX10 inverted microscope and the ZEN imaging software, and left for an additional period of three days. Each well was reexamined each day for a period of 14 days to determine the level of confluence and the presence of resistant cells.

For detection of apoptosis and necrosis, 48 h after adhesion, the medium was replaced with fresh medium containing 5 μM YO-PRO-1 (Molecular Probes, Eugene, OR), 20 μM propidium iodide (Molecular Probes), and either 10 μM cisplatin, 10 μM SAHA or increasing concentrations of curcumin (10, 25, 50, 75 or 100 μM), and the 12-well plate was placed under a Zeiss Axio Observer.Z1 microscope (Carl Zeiss MicroImaging GlbH, Germany) equipped with an environmental chamber maintained at 37°C and 5% CO_2_ and an ORCA Flash4.0 v2 camera (Hamamatsu Photonics, Hamamatsu City, Japan) and driven by Metamorph software (Molecular Devices, Downingtown, PA). Fluorescent images at a 10-fold magnification of cells stained with YO-PRO-1 and propidium iodide were successively recorded every 10 min during 24 h.

### Histopathology and immunohistochemistry

Tumor and tissue samples from the liver, spleen, pancreas, gut, diaphragm, mesenteric lymph nodes, parietal peritoneum and adipose tissue were fixed in 4% buffered paraformaldehyde, embedded in paraffin wax, cut with a Bond Max automaton (Menarini, Rungis, France) and stained with hematoxylin-phloxine-saffron (HPS). Antibodies used for immunohistochemical analyses were anti-rat ED1 antibody (MAB1435 1/100, EMD Millipore Corporation, Billerica, MA, USA) used as a pan-macrophage marker, mouse anti-CD3 (SM253P, Acris Antibodies, San Diego, USA), anti-CD8 (LS-B3665, LSBio France, 92000 Nanterre), mouse anti-rat IL-6 (AT-3060, Clinisciences, Nanterre, France), and anti-vimentin (ab8978, Abcam), with an anti-mouse secondary antibody and N-Histofine Simple Stain Mouse MAX Peroxidase (Nichirei Biosciences, Tokyo, Japan) as the detection reagent. Histopathology slides were scanned with a Nanozoomer 2.0 HT (Hamamatsu, Japan).

### *In vivo* studies

Three successive *in vivo* experiments were conducted in this study. In the first experiment (E1), a group of three male and three female F344 rats was used to determine the potential of the M5-T1 mesothelioma cell line to produce tumors in syngeneic rats three weeks after i.p. transplantation of 5 × 10^6^ cells into the peritoneal cavity. The second experiment (E2) was aimed at comparing the potential of curcumin or SAHA injected i.p. to kill M5-T1 cells *in vivo*. For this purpose, two successive injections of 750 μg/kg curcumin (3 rats) or 50 mg/kg SAHA (3 rats) were given i.p. to young male F344 rats (8 weeks of age) at days 21 and 26 after tumor challenge (day 0), and the animals were euthanized at day 28. Three other rats received only the vehicle (DMSO, 50 μl in 0.5 ml saline, i.p.) and served as controls (euthanized at day 28). To optimize the curcumin treatment procedure and to simulate the conditions of use in patients, a third experiment (E3) was conducted on 12 older male rats (16 weeks of age). In order to follow the recommendations on the welfare and use of animals in cancer research, particular attention was given in this experiment to incorporating the objectives of the 3Rs (replacement, reduction and refinement) [21]. In particular, given the knowledge of the pathogenesis of this model collected in experiments E1 and E2, the number of M5-T1 cells injected for tumor challenge was limited to 3 x10^6^ cells and a shorter-duration endpoint was chosen for euthanasia (day 16), in order to limit the consequences of tumor burden to the animals. After challenging the rats with M5-T1 cells (on day 0), four successive injections of 1.5 mg/kg curcumin (corresponding to 150 μl of the 10 mM stock solution diluted in 0.3 ml saline) were given i.p. to six rats on days 7, 9, 11 and 14. The six other rats received only the vehicle (DMSO, 150 μl in 0.3 ml saline) and served as controls. All rats were euthanized at day 16, and all tissues including small metastatic nodules or residual tumor tissue were collected and fixed for further determination of total tumor masses and histological examination.

## SUPPLEMENTARY MATERIALS FIGURES AND TABLES


