# Supplementary material for: Evaluation of intracavitary administration of curcumin for the treatment of sarcomatoid mesothelioma
Source: Oncotarget. 2017 Feb 25;8(34):57552–73. doi: 10.18632/oncotarget.15744 (PMC5593667; doi:10.18632/oncotarget.15744)
Supplement: Supplementary file 1 [file oncotarget-08-57552-s001.pdf]

Evaluation of intracavitary administration of curcumin for the treatment of sarcomatoid mesothelioma

Supplementary Material

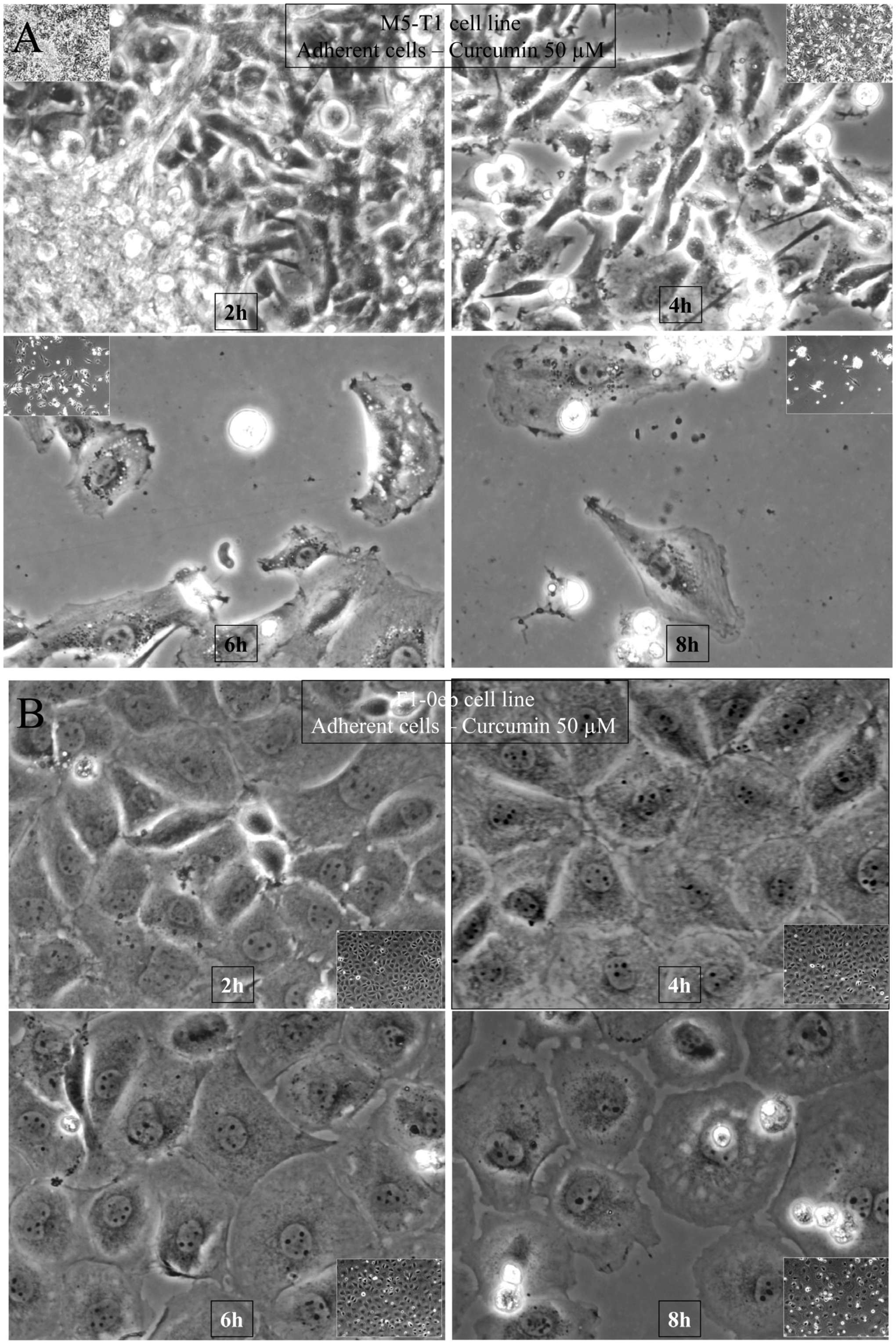

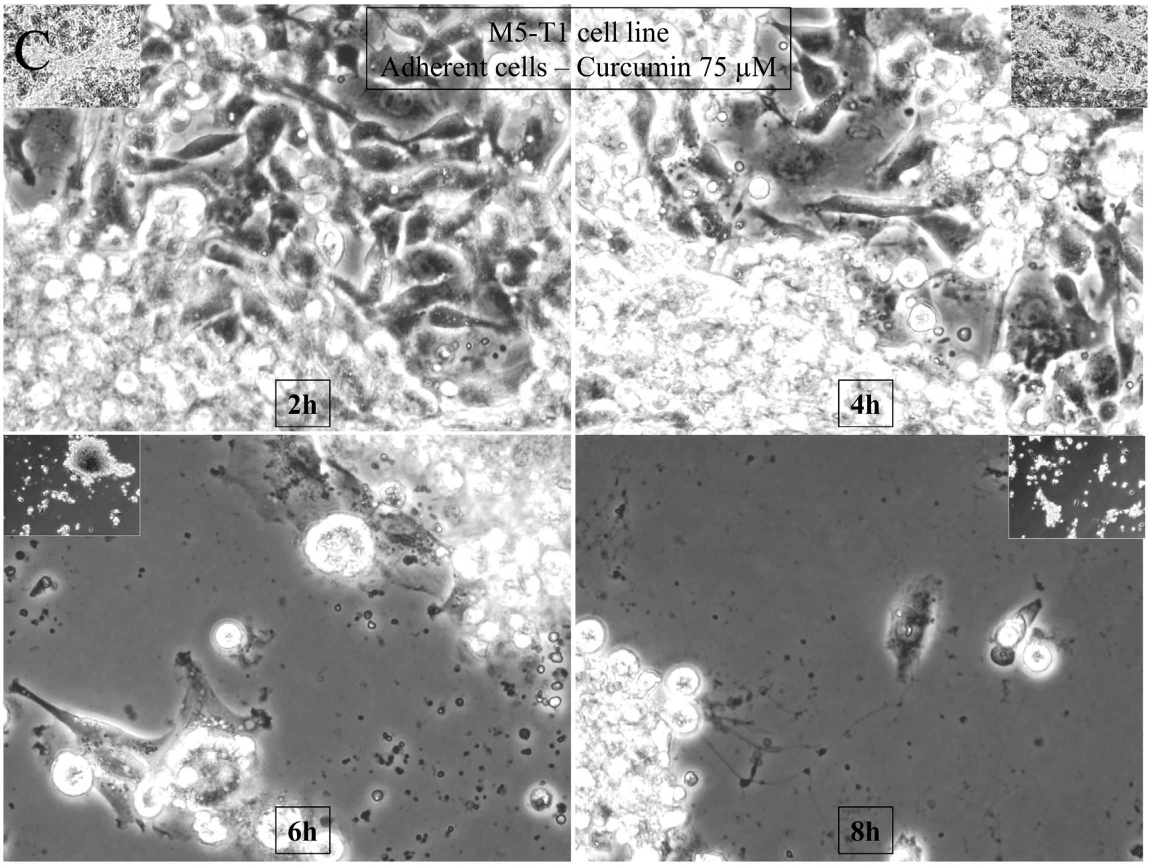
C
M5-T1 cell line
Adherent cells – Curcumin 75 μM
2h
4h
6h
8h

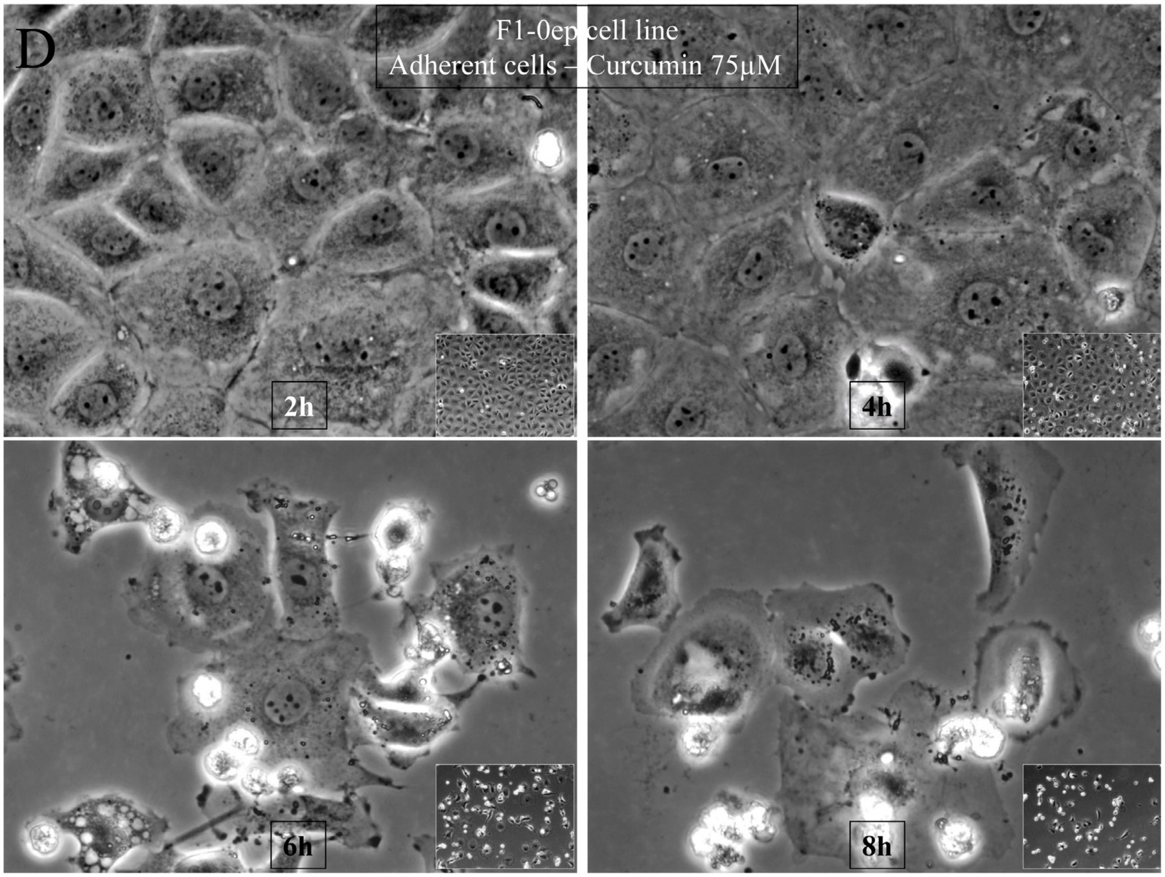
D
F1-0ep cell line
Adherent cells – Curcumin 75μM
2h
4h
6h
8h

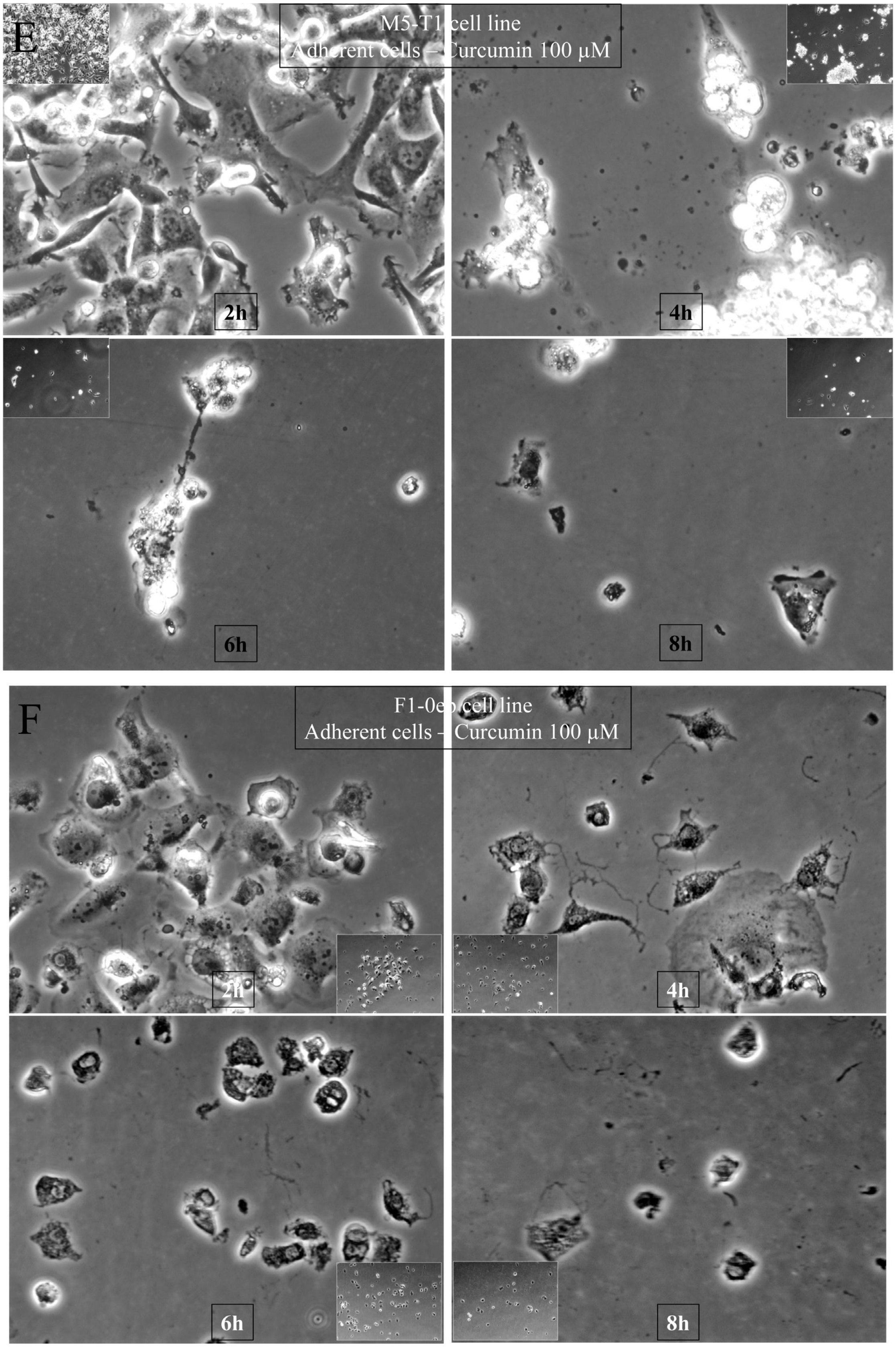

Supplemental Fig. S1. Concentration and time dependency of M5-T1 and F1-0ep killing by curcumin (adherent cells). Photomicrographs of adherent M5-T1 cells (A, C and E) and F1-0ep cells (B, D and F) at day 2 after curcumin treatment, x320. Inserts show general views, x100.

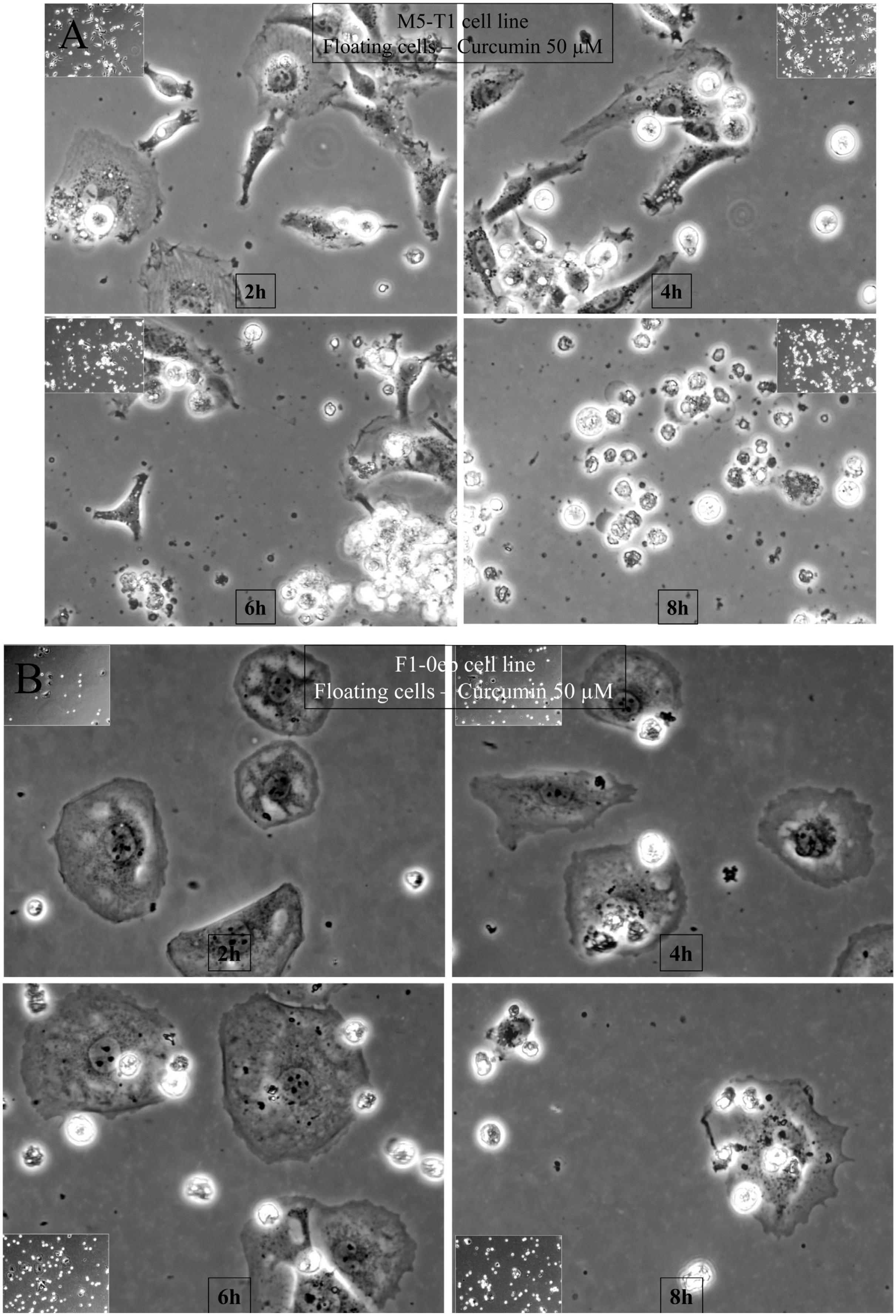
A
M5-T1 cell line
Floating cells – Curcumin 50 μM
2h
4h
6h
8h
B
F1-0ep cell line
Floating cells – Curcumin 50 μM
2h
4h
6h
8h

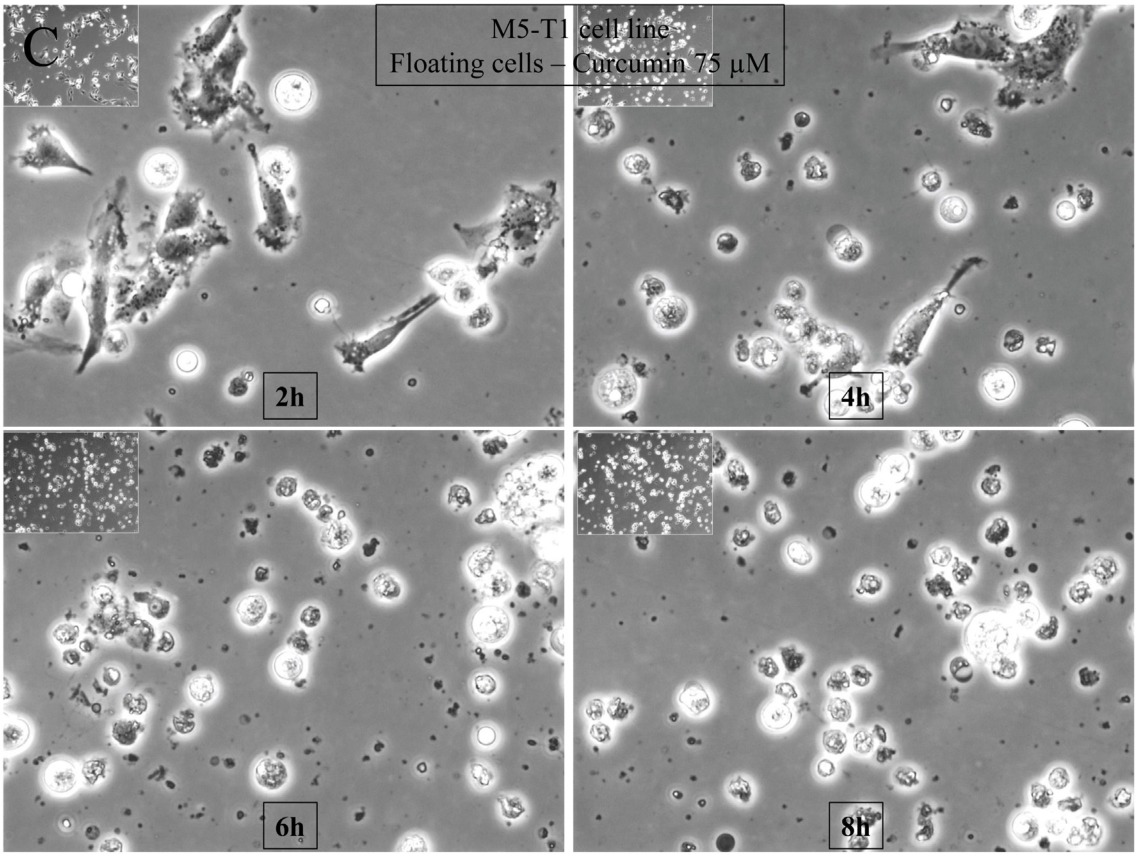
C
M5-T1 cell line
Floating cells – Curcumin 75 µM
2h
4h
6h
8h

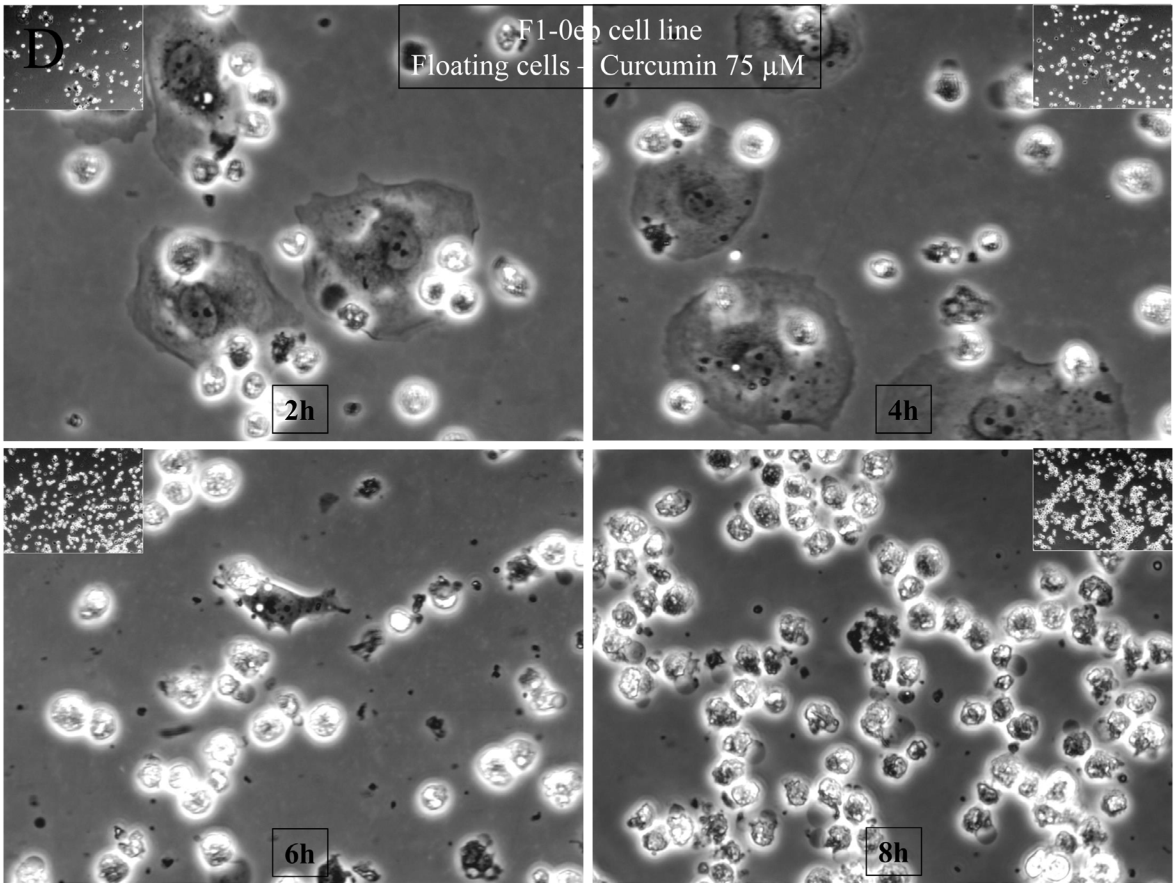
D
F1-0eb cell line
Floating cells – Curcumin 75 µM
2h
4h
6h
8h

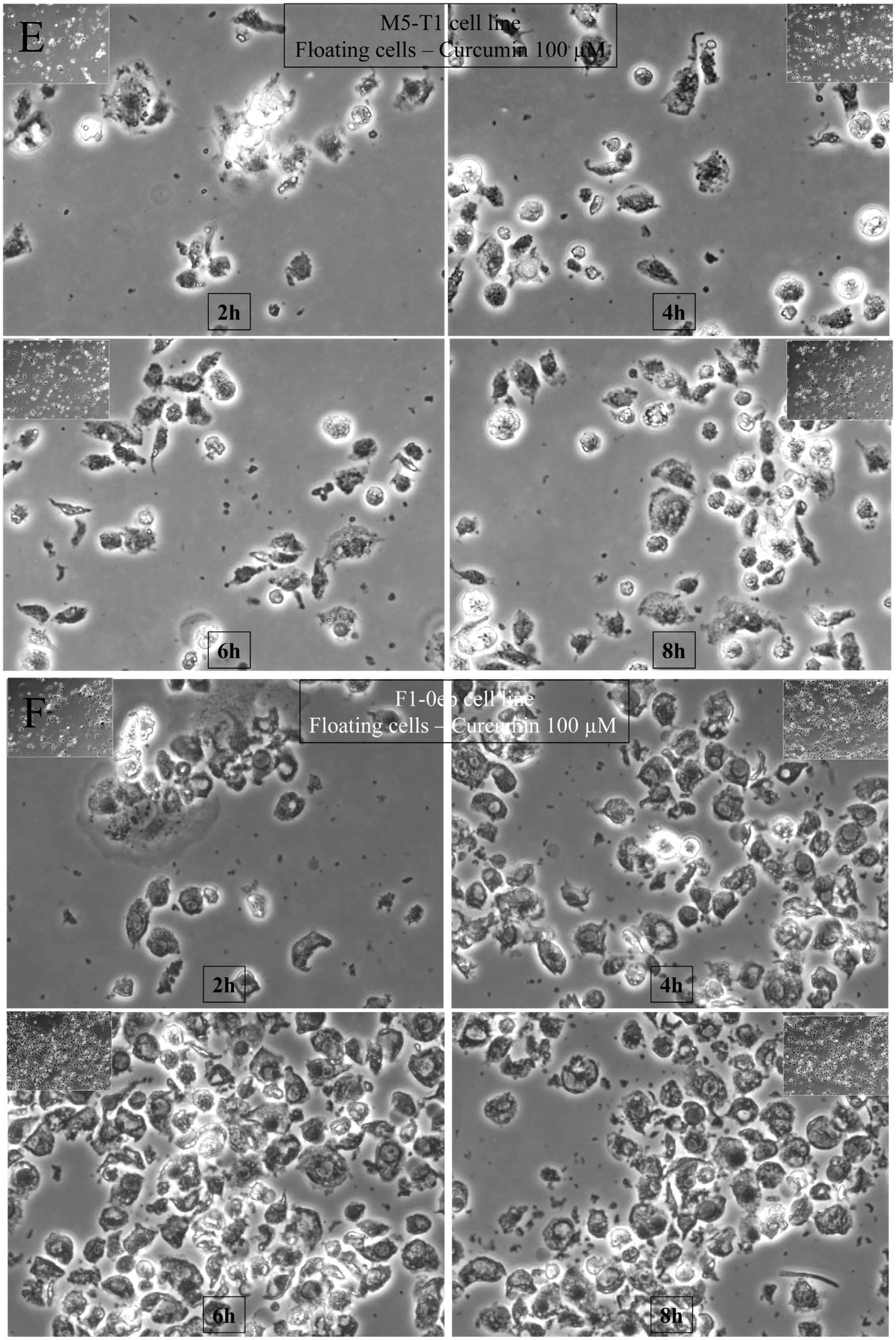

Supplemental Fig. S2. Concentration and time dependency of M5-T1 and F1-0ep killing by curcumin (floating cells). Photomicrographs of floating M5-T1 cells (A, C and E) and F1-0ep cells (B, D and F) at day 2 after curcumin treatment (cells resuspended and transferred into a new 12-well plate at day 1), x320. Inserts show general views, x100.

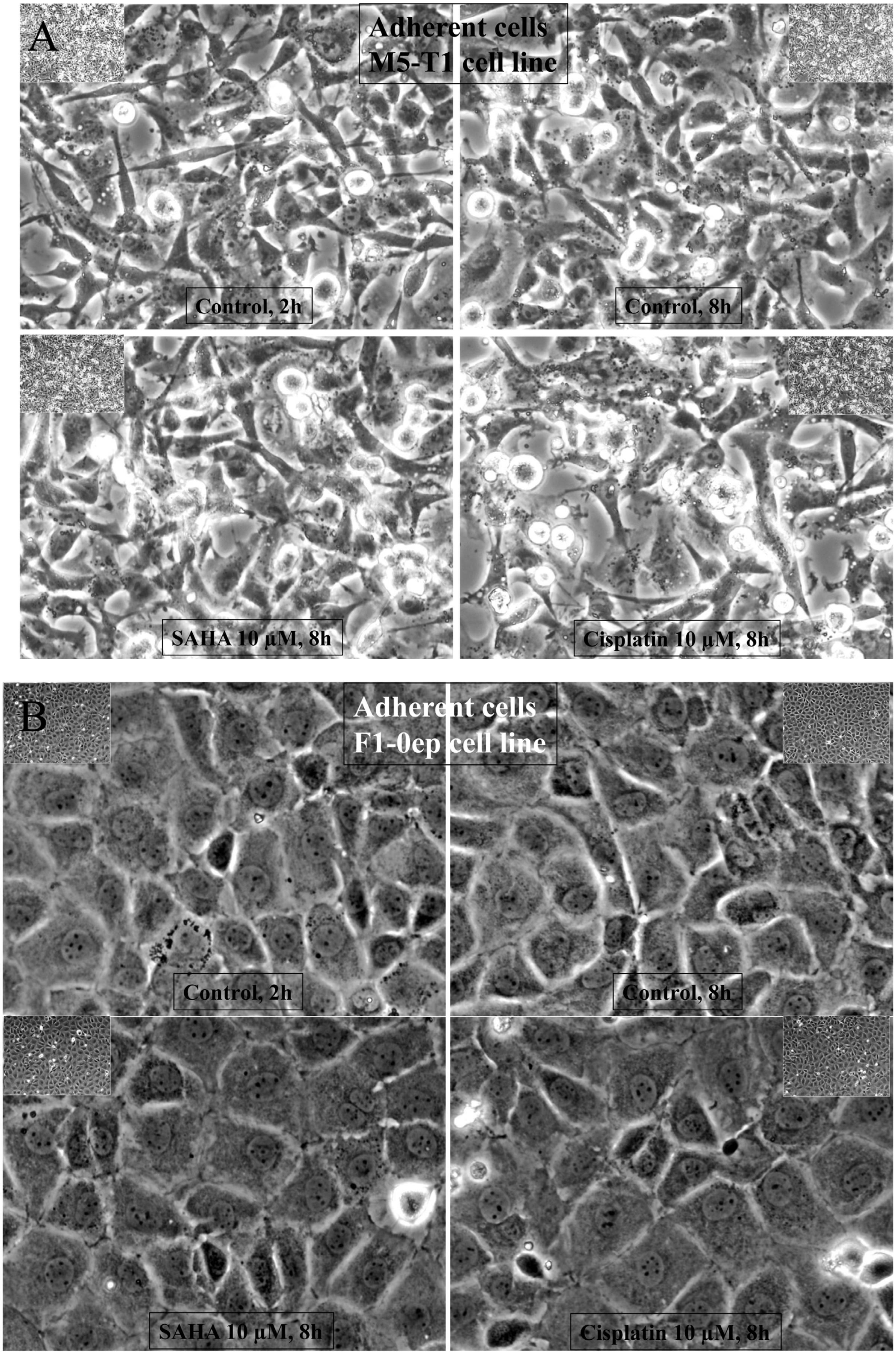
A
Adherent cells
M5-T1 cell line
Control, 2h
Control, 8h
SAHA 10 µM, 8h
Cisplatin 10 µM, 8h
B
Adherent cells
F1-0ep cell line
Control, 2h
Control, 8h
SAHA 10 µM, 8h
Cisplatin 10 µM, 8h

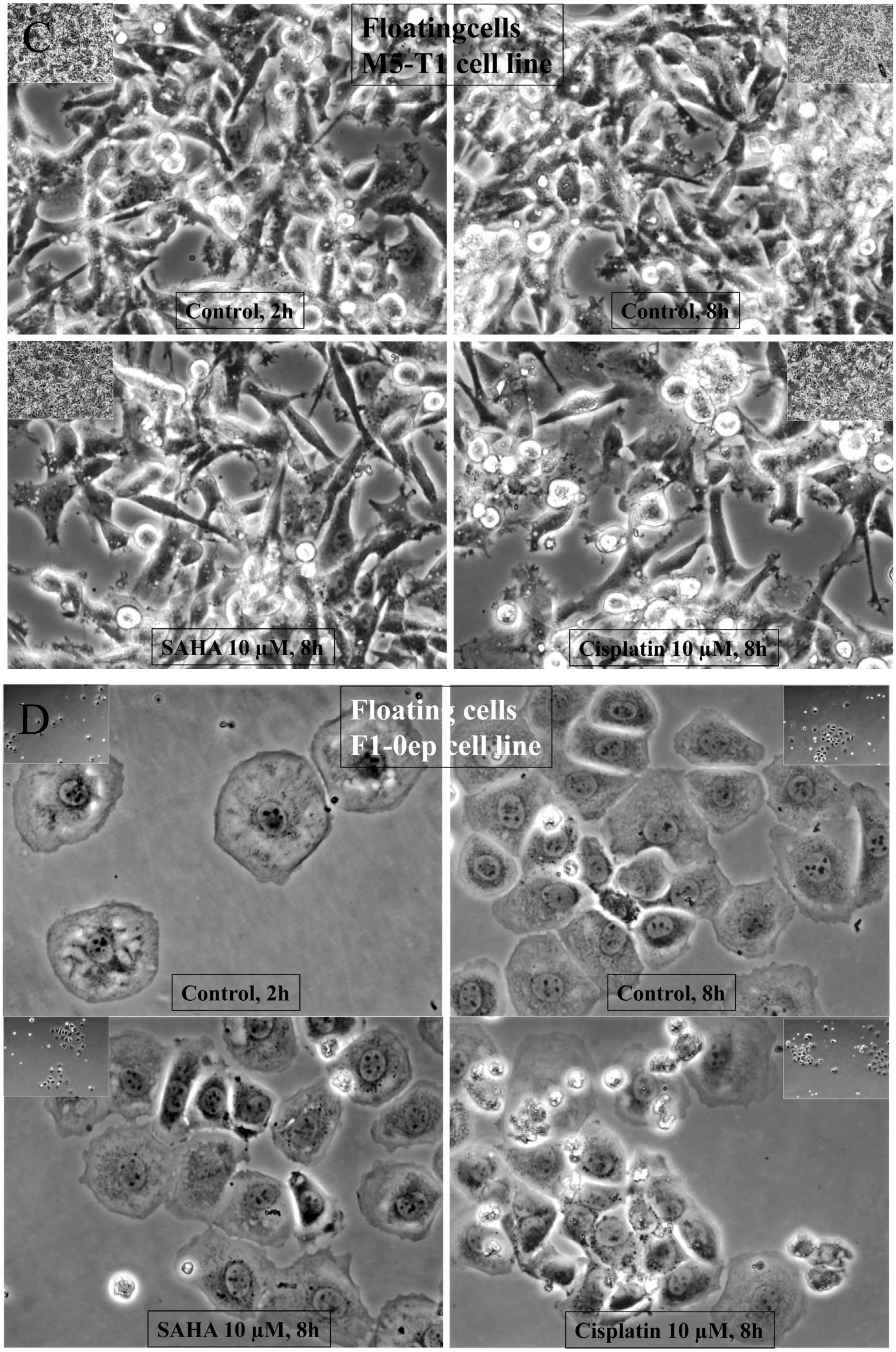

Supplemental Fig. S3. Concentration and time dependency of M5-T1 and F1-0ep treatment by SAHA or cisplatin. Photomicrographs of adherent (A, B) and floating (C, D) M5-T1 cells (A, C) and F1-0ep cells (B, D) at day 2 after treatment with 10 µM SAHA or 10 µM cisplatin, in comparison with control cells (incubation with normal medium containing 1% DMSO), x320. Inserts show general views, x100. Photomicrographs of intermediate incubation times were excluded when they showed comparable situations.

A

Curcumin

HPS

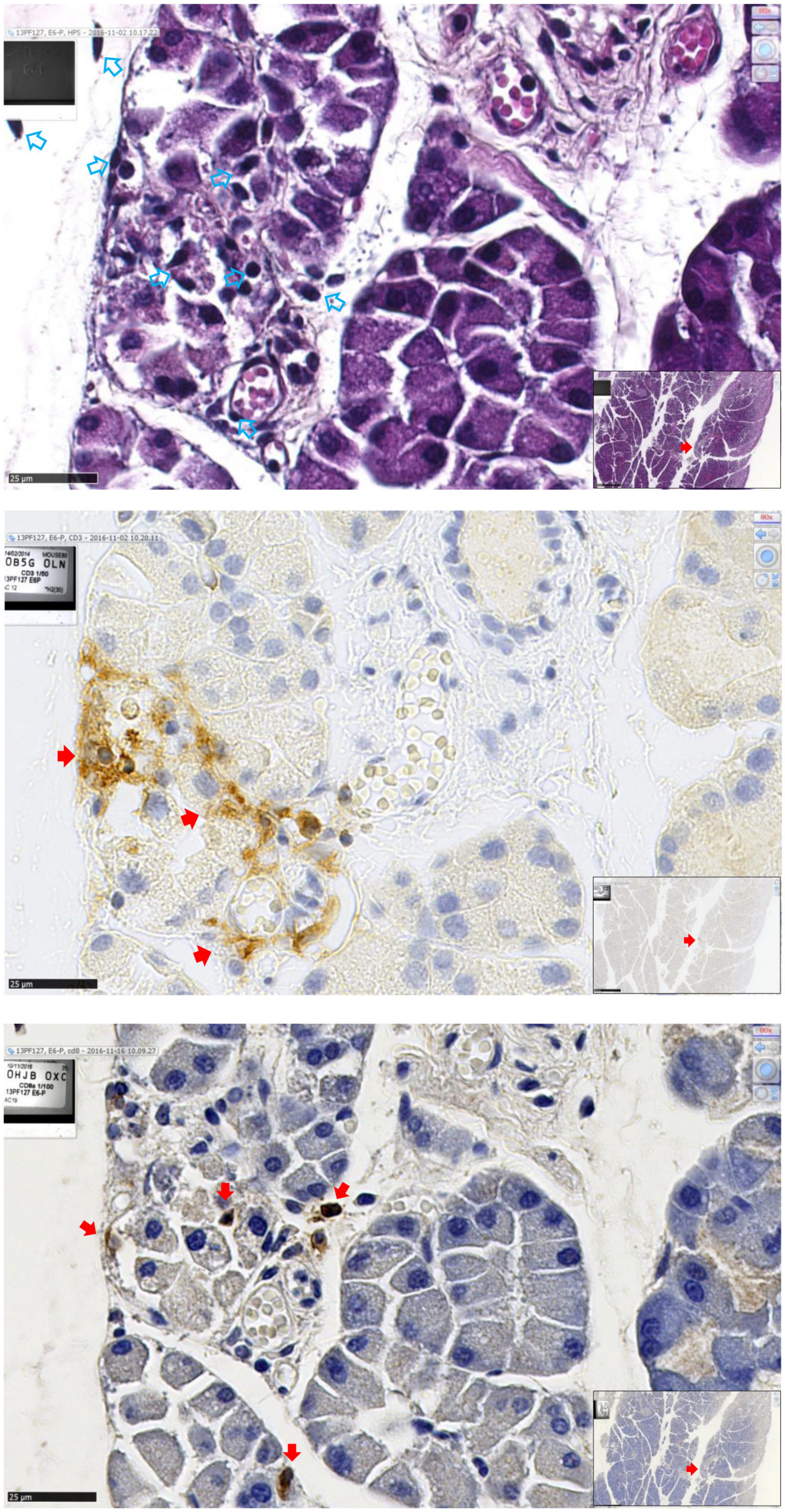

B **Control (no treatment)**

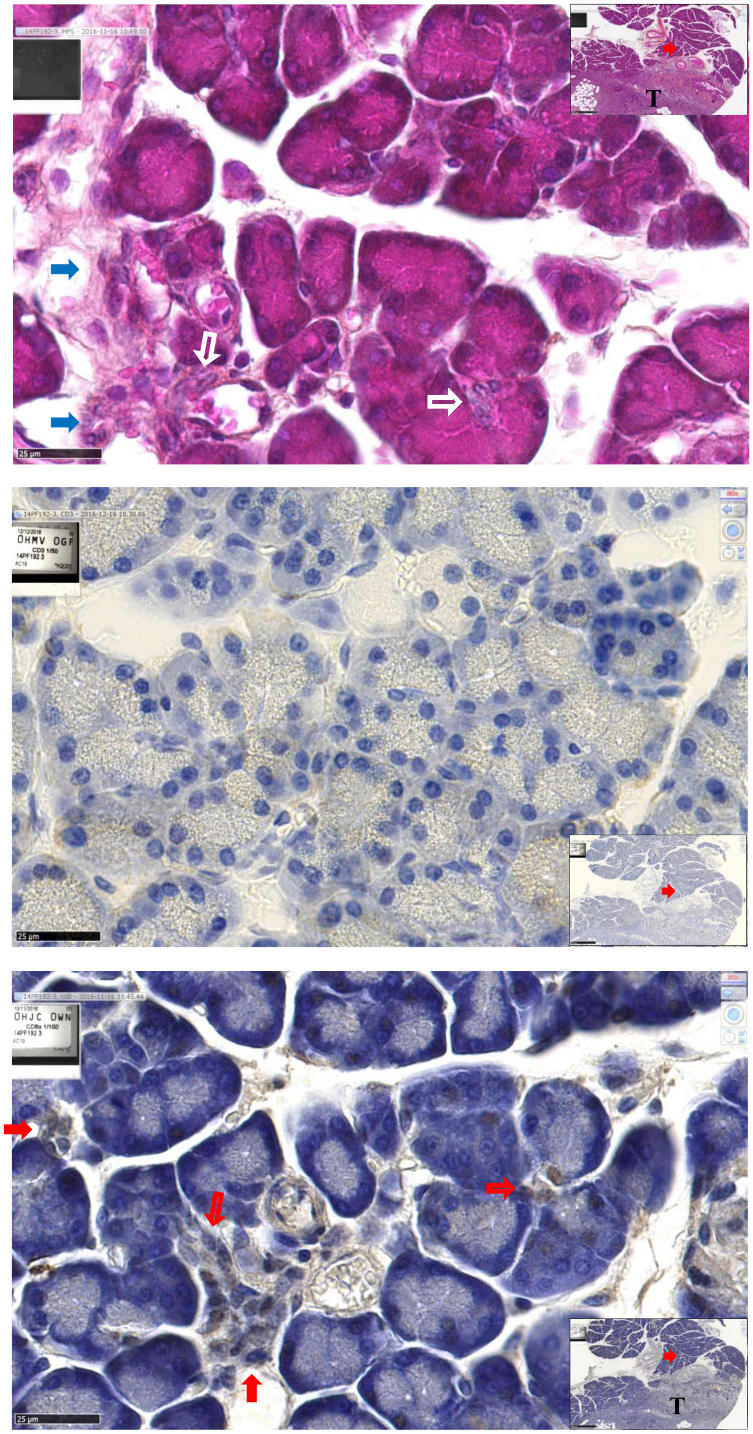

Supplemental Fig. S4. Presence of circulating CD8+ T lymphocytes in the pancreas of rats treated with curcumin, comparison with control (untreated) rats. (A) Representative example of the subgroup of rats treated with curcumin, indicated by a red arrow in Fig. 9A. Large views, x 800, the scale bars represent 25 µm. Inserts show general views (x 50), arrows indicating the localization of magnifications, scale bars represent 500 µm. Top, HPS, the blue arrows show numerous cells densely stained with a very high nucleus to cytoplasm ratio, suggesting lymphocytes. Middle and bottom, immunohistochemical stainings of the same area with monoclonal antibodies against CD3 and CD8, respectively. Red arrows confirm the presence of T lymphocytes and CD8+ positive cells. (B) Representative example of the group of control (untreated) rats with a marked invasion of the pancreas by M5-T1 tumor cells. Top, HPS, the blue or white arrows show numerous M5-T1 tumor cells invading the space between pancreatic acini. Middle and bottom, immunohistochemical stainings of the same area with monoclonal antibodies against CD3 and CD8, respectively, showing the absence of T lymphocytes and CD8+ cells. Red arrows show the localization of tumor cells.

A

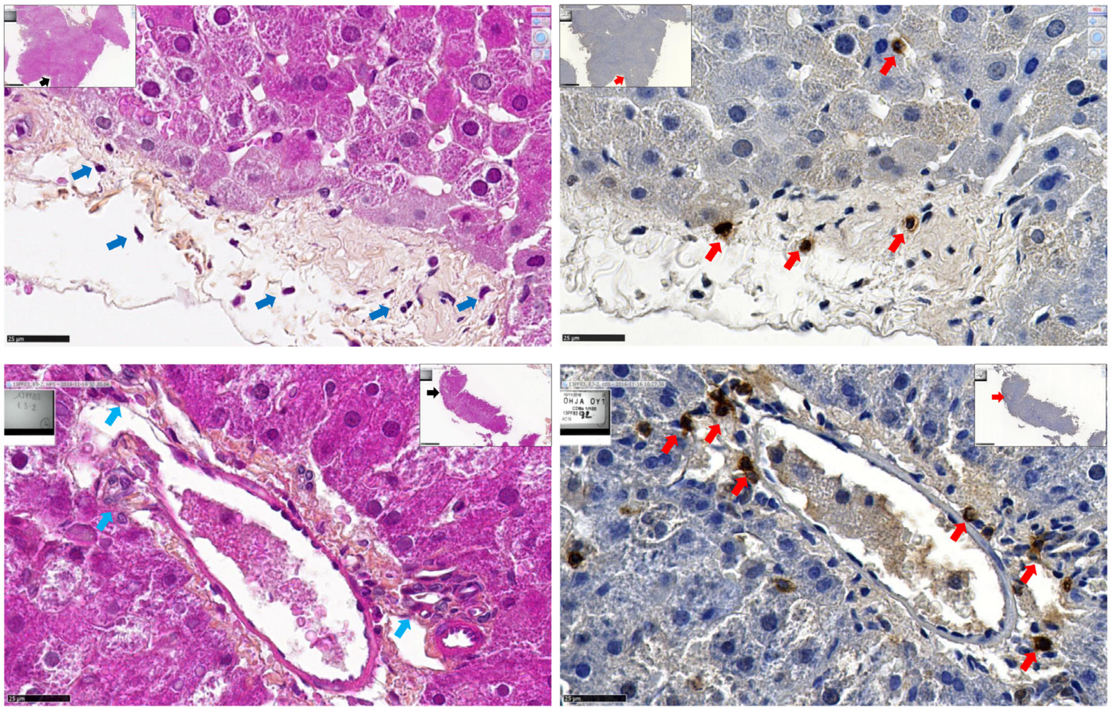

B

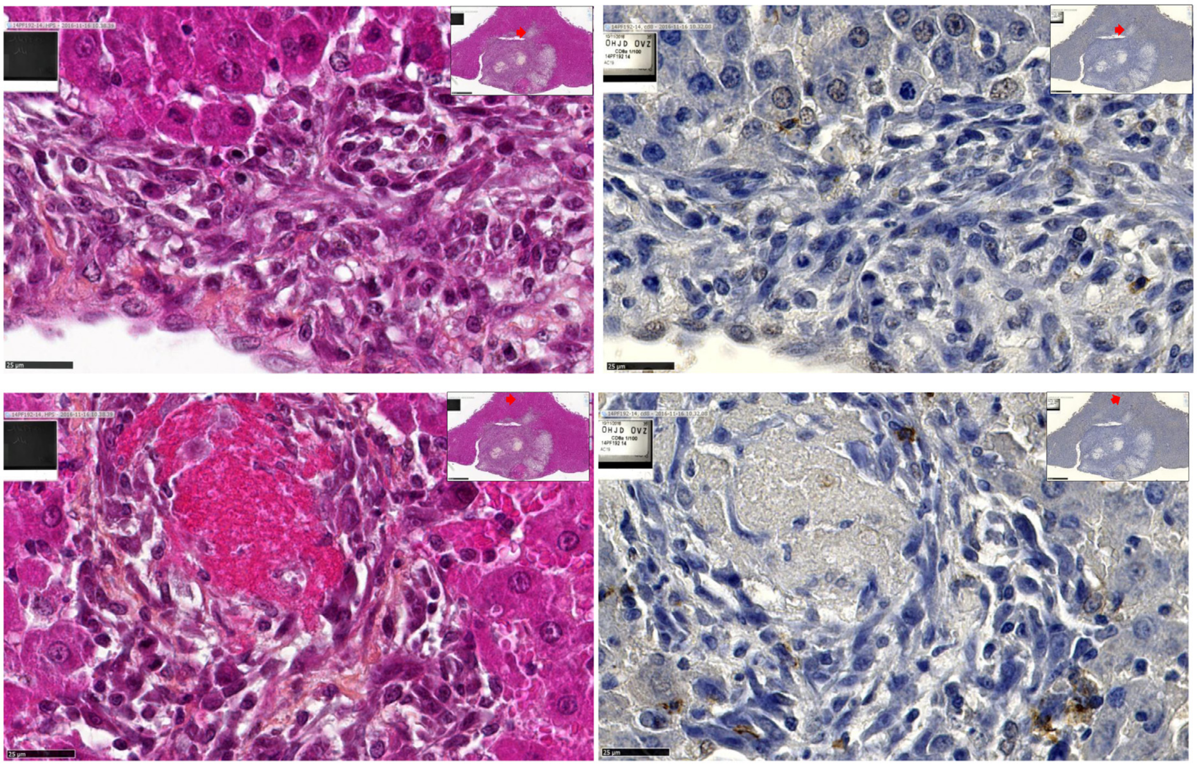

C

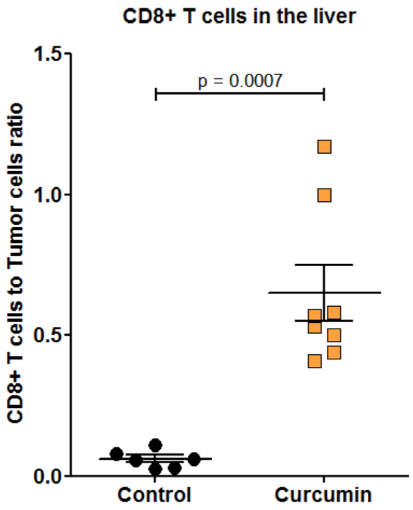

Supplemental Fig. S5. Presence of circulating CD8+ T lymphocytes in the liver of rats treated with curcumin, comparison with control (untreated) rats. (A), Two representative examples from the subgroup of rats treated with curcumin, indicated by a red arrow in Fig. 9A. Large views, x800, the scale bars represent 25 µm. Inserts show general views (x50), arrows indicating the localization of magnifications, scale bars represent 500 µm. Top, HPS (left) and immunohistochemical staining with anti-CD8 monoclonal antibody (right) of the liver capsule showing numerous cells (blue arrows) with the same characteristics as in supplemental Fig. S4-A (top), and the presence of CD8+ T lymphocytes (red arrows). Bottom, Images of a liver venule showing the presence of CD8+ T lymphocytes (immunohistochemical staining with anti-CD8 monoclonal antibody, right) in contact with some isolated M5-T1 tumor cells (HPS, left). (B), Two representative examples of the group of control (untreated) rats showing a dramatic invasion of the liver capsule (top) and a liver venule (bottom) by M5-T1 tumor cells, and very rare CD8+ T lymphocytes. Left column, HPS; right column, immunohistochemical staining with anti-CD8 monoclonal antibody. (C), Quantification of the number of CD8+ T lymphocytes in areas of hepatic veins.

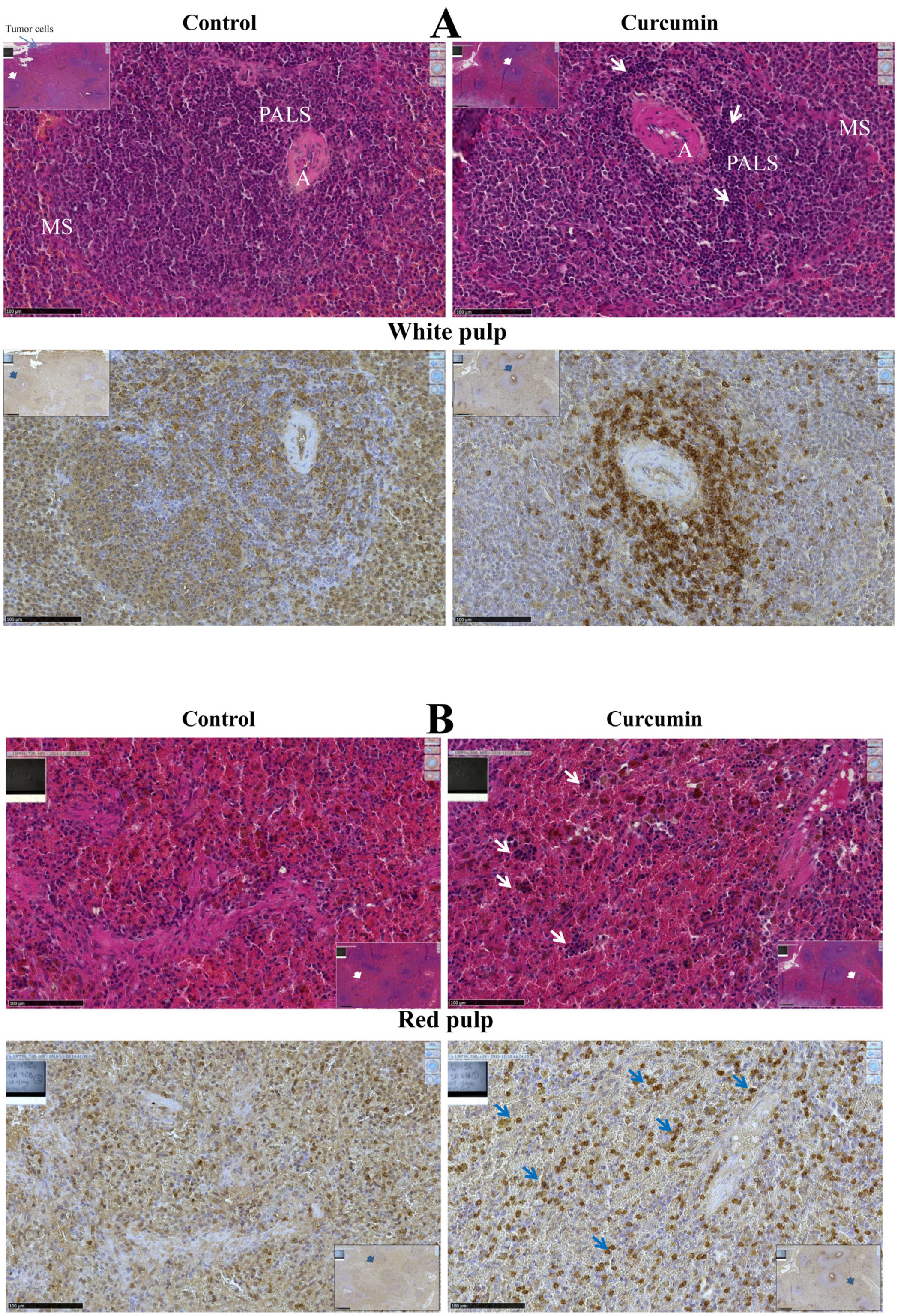

Supplemental Fig. S6. Changes induced in CD8+ T lymphocytes of the spleen by curcumin treatment. (A), White pulp, representative rats from the control (untreated) group (left column) and the subgroup of rats treated with curcumin, indicated by a red arrow in Fig. 9A (right column). Top images, HPS staining. The white arrows indicate the localization of dense foci of CD8+ T lymphocytes revealed after immunohistochemical staining with anti-CD8 monoclonal antibody (bottom images). PALS, periarteriolar lymphoid sheath; A, central artery; MS, marginal sinus. x800, the scale bars represent 25 µM. Inserts show general views (x50), arrows indicate the localization of magnifications, scale bars represent 500 µm. (B), Red pulp of the same rats. The white (top) and blue arrows (bottom) indicate the localization of CD8+ T lymphocytes revealed after immunohistochemical staining with anti-CD8 monoclonal antibody (bottom images).
